# Methylation Drives SLC2A1 Transcription and Ferroptosis Process Decreasing Autophagy Pressure in Colon Cancer

**DOI:** 10.1155/2022/9077424

**Published:** 2022-08-27

**Authors:** Junwei Zou, Zhenhan Li, Jiaheng Xie, Zhaoying Wu, Yujin Huang, Hao Xie, Huiqiu Xu, Yong Huang, Hailang Zhou

**Affiliations:** ^1^Department of Gastrointestinal Surgery, the Second Affiliated Hospital of Wannan Medical College, Wuhu, Anhui, China; ^2^School of Clinical Medicine, Wannan Medical College, Wuhu, Anhui, China; ^3^Department of Burn and Plastic Surgery, the First Affiliated Hospital of Nanjing Medical University, Nanjing, Jiangsu, China; ^4^School of Pharmacy, Wannan Medical College, Wuhu, Anhui, China; ^5^Department of Gastroenterology, Lianshui People's Hospital, Kangda College of Nanjing Medical University, Huai'an, Jiangsu, China

## Abstract

Colon cancer is a common malignant tumor in the digestive tract, with relatively high rates of morbidity and mortality. It is the third most common type of tumor in the world. The effective treatment of advanced colon cancer is limited, so it is particularly important to study the new pathogenesis of colon cancer. Ferroptosis is a nonapoptotic regulated cell death mode driven by iron-dependent lipid peroxidation, a process which has been discovered in recent years. Autophagy involves lysosomal degradation pathways that promote or prevent cell death. High levels of autophagy are associated with ferroptosis, but a clear association has not yet been made between ferroptosis and autophagy in colon cancer. Through the analysis of transcriptome expression profiling data in colon cancer, we obtained the common upregulated genes and downregulated genes by recording the intersection of the differentially expressed genes in each dataset. Solute Carrier Family 2 Member 1 (SLC2A1) was identified by combining autophagy genes obtained from GeneCards and ferroptosis genes obtained from FerrDb. In order to explore the clinical significance and prognostic value of SLC2A1, we utilized massive databases to conduct an in-depth exploration of the methylation of SLC2A1, and we also investigated the differences in immune infiltration between tumor and normal tissues. We found that there are abundant methylation sites in SLC2A1 and that the methylation of SLC2A1 is correlated with the immunosuppression of tumor tissue. We discovered that during the induction of environmental factors, the transcription and methylation levels of SLC2A1 were greatly increased, autophagy and ferroptosis were inhibited, and the immune system was defective, resulting in a poor prognosis for patients. These results suggest that the autophagy and ferroptosis-related gene SLC2A1 is involved in the tumor immune regulation of colon cancer, and SLC2A1 may become a new therapeutic target for colon cancer.

## 1. Introduction

Cancer is a chronic disease characterized by abnormal cell growth that gradually spreads and destroys normal body tissues as the cancer progresses [1]. It is one of the most prominent life-threatening diseases worldwide. According to the latest estimates from the International Agency for Research on Cancer (IARC), new cancer cases will continue to increase. Colon cancer is the third most common malignant tumor in the world, the second most common cancer among females, and the third most common cancer among males, with 1.2 million new cases and more than 600,000 deaths. Survival rates have improved over the past three decades due to early detection, but the 5-year survival rate for patients diagnosed at a later stage of the disease is still only 14% [2–5]. Colon cancer remains a relatively difficult disease to treat, although advanced treatments are available which can improve survival rates for the disease. Therefore, it is particularly important to conduct an in-depth exploration of the pathogenesis of colon cancer and new therapeutic targets.

Ferroptosis is a nonapoptotic regulated cell death mode driven by iron-dependent lipid peroxidation, a process which has been discovered in recent years. Ferroptosis has been reported to be involved in the pathogenesis of colon cancer [6, 7]. The induction of ferroptosis is accompanied by an increase in intracellular lipids and reactive oxygen species (ROS), leading to cell death, which is why lipid antioxidants can inhibit ferroptosis. Since iron is an essential ion in the mitochondrial oxidative respiratory chain, mitochondria, as organelles that are rich in iron ions and which generate ROS, are considered to be an important site for ferroptosis. Not only are they an important site for intracellular ROS generation, but fatty acid metabolism provides specific lipid precursors for cellular ferroptosis. Autophagy, which refers to any intracellular process involving lysosome degradation of cytosolic components, also occurs during tumor progression [8]. Autophagy is essential for cell survival, differentiation and development, and homeostasis [9]. Autophagy is involved in a variety of diseases, including infection, cancer, neurodegeneration, and ageing, as well as heart, liver, and kidney disease [10]. When ferroptosis occurs, cells will face additional pressures such as hypoxia, oxidative stress, and oncogene activation, which may induce autophagy. In mitochondria, ferroptosis results in decreased mitochondrial volume and cristae and increased membrane density. This can also cause cells to erroneously and selectively phagocytose mitochondria. In conclusion, there may be an association between ferroptosis and autophagy in colon cancer, and the mechanism of their interaction has not yet been explored.

In this study, we attempted to explore the mechanism of the interaction between ferroptosis and autophagy in colon cancer. By analyzing three different transcriptome datasets of colon cancer patients, we obtained the common upregulated genes and downregulated genes by taking the intersection of differentially expressed genes. The gene SLC2A1 was identified by combining autophagy genes obtained from GeneCards and ferroptosis genes obtained from FerrDb. To explore the clinical significance and prognostic value of SLC2A1, we used massive databases to explore in depth the methylation of SLC2A1, and we investigated the differences in immune infiltration between tumor and normal tissues using the gene expression omnibus (GEO) dataset. We found that there are a lot of methylation sites in SLC2A1 and that the methylation of SLC2A1 is correlated with the immunosuppression of tumor tissue. Our results suggest that SLC2A1 further modulates the immune microenvironment by regulating ferroptosis and autophagy processes in colon cancer, thereby affecting disease prognosis.

## 2. Materials and Methods

### 2.1. Data Acquisition

GEO is the world's largest public database containing genetic data on various types of diseases [11]. We downloaded the expression profile data of GSE10972, GSE23878, and GSE113513 for colon cancer. Data processing was performed using R software to obtain differential genes.

### 2.2. Information of Patients

We downloaded the data from the Cancer Genome Atlas (TCGA) for various analyses, including the differential expression analysis of unpaired samples, the differential expression analysis of paired samples, construction of the survival curve and receiver operating characteristic (ROC) curve , and the correlation analysis of the DNA methylation probe and expression level. The differential expression analysis of unpaired samples and the data of the ROC curve were all derived from the RNAseq data in Transcripts Per Kilobase of exon model per million mapped reads (TPM) format from the TCGA and the Genotype-Tissue Expression (GTEx) databases, processed by UCSC Xena (https://xenabrowser.net/datapages/) through the Toil process. We extracted the corresponding normal tissue data from colon adenocarcinoma (COAD) and GTEx of TCGA, and then the log2 normalization was used to transform the RNAseq data in TPM format. The paired samples and survival curve data were derived from RNAseq data in the level 3 HTSeq-Fragments Per Kilobase per Million (FPKM) format in the TCGA (https://portal.gdc.cancer.gov/) COAD portal. We converted the RNAseq data in FPKM format into TPM format and performed a log2 normalization.

### 2.3. Survival Curve Analysis

The principal R package used to express the difference was ggplot2 [version 3.3.3] (for visualization). The R packages used in the ROC curve were pROC [1.17.0.1 version] (for analysis) and ggplot2 [3.3.3 version] (for visualization), with the false positive rate (FPR) on the abscissa and the true positive rate (TPR) on the ordinate. The survival curve used the survminer [version 0.4.9] (for visualization) and survival [version 3.2–10] (for statistical analysis of survival data) R packages. The types of prognosis included overall survival (OS) and progression-free interval (PFI). Significance mark: ns, *p* ≥ 0.05; ^*∗*^*p* < 0.05; ^*∗∗*^*p* < 0.01; ^*∗∗∗*^*p* < 0.001.

### 2.4. PPI Analysis

We used the STRING (https://cn.string-db.org/) website, which contains data of many species [12] relating to protein-protein interaction networks and functional enrichment analysis. We entered the details of the intersection genes, obtained the protein interaction network, and downloaded the protein interaction data for subsequent analysis. We used the GeneMANIA (https://genemania.org/) website to help us predict the biological functions of specific genes and gene sets [13]. Cytoscape is a software application that visualizes molecular interaction networks and biological pathways and integrates these networks with annotations, gene expression profiles, and other status data [14]. In Cytoscape (https://cytoscape.org/), we imported the protein-protein interaction networks of 186 genes obtained from STRING to remove the unconnected proteins and then used the cytoHubba plug-in to calculate and sequence the MCCs.

### 2.5. Harvesting of Functional Gene Lists: FerrDb and GeneCards

GeneCards (https://www.genecards.org/) is a comprehensive database comprising information about genes, including details of genomes, transcriptomes, and proteins, as well as genetic, clinical, and functional aspects of gene-centric data. We downloaded 6866 genes related to autophagy using “autophagy” as the keyword. The autophagy gene was intersected with the differential gene in GEO.

FerrDb (https://www.zhounan.org/ferrdb/legacy/index.html) was the first comprehensive database containing the genes proven to be related to ferroptosis; it also includes a number of monomers that can regulate ferroptosis [15]. We obtained 260 genes related to ferroptosis, including driver, marker, and suppressor genes.

### 2.6. Bioinformatic Processing

#### 2.6.1. EWAS

The EWAS (https://ngdc.cncb.ac.cn/ewas/datahub) database contains a large amount of data of disease types and was therefore convenient for helping us to visualize methylation levels [16]. We were able to determine the relationship between the methylation status of SLC2A1 in COAD and the methylation level and prognosis.

#### 2.6.2. UALCAN

UALCAN (https://ualcan.path.uab.edu/index.html) is a comprehensive, user-friendly tumor database that provides users with a convenient way to explore TCGA, the National Cancer Institute's Clinical Proteomic Tumor Analysis Consortium (CPTAC), and other datasets [17]. From this database, we obtained methylation data (from TCGA) and protein data (from CPTAC) for SLC2A1.

#### 2.6.3. The Human Protein Atlas (HPA)

This is a unique website dedicated to providing immunohistochemical images, including those of many kinds of tumors and corresponding normal tissues; it proved useful to us for exploring the protein content of genes (https://www.proteinatlas.org/) [18]. We obtained high-resolution immunohistochemical images of normal tissues and tumor tissues from the website to determine the abundance of protein.

#### 2.6.4. CIBERSORTx

This was used to estimate the gene expression profile and provide the estimated abundance of member cell types in the mixed cell population by using the gene expression data (https://cibersortx.stanford.edu/) [19]. We cleaned the original expression data from GEO, with the requirements that the expression values do not undergo log conversion and cannot have null values.

#### 2.6.5. EcoTyper

EcoTyper (https://ecotyper.stanford.edu/) is a new machine learning framework for the identification of cell states and ecosystems from bulk, single-cell, and spatially-resolved expression data [20]. EcoTyper extends CIBERSORTx for large-scale profiling of cellular ecosystems.

#### 2.6.6. xCell

xCell (https://xcell.ucsf.edu/) is a webtool that performs cell type enrichment analysis from gene expression data for 64 immune and stroma cell types [21]. xCell is a gene signature-based method learned from thousands of pure cell types from various sources. In order to explore the tumor heterogeneity of colon cancer, we inferred the types of sample cells from the expression profile in xCell.

#### 2.6.7. MEXPRESS

This is a user-friendly database that visualizes DNA methylation, expression, and clinical data (https://www.mexpress.be/) [22].

#### 2.6.8. MethSurv

This is a web tool for performing multivariate survival analysis using DNA methylation data (https://biit.cs.ut.ee/methsurv/) [23]. We used this website to explore the relationship between methylation levels of different methylation probes and prognosis.

#### 2.6.9. SRAMP

SRAMP is a sequence-based RNA adenosine methylation site predictor; it is used to predict the m6A modification site on the target mRNA sequence (https://www.cuilab.cn/sramp) [24].

#### 2.6.10. JASPAR

This database contains a large amount of transcription factor data, which can help researchers to predict the binding sites of transcription factors and their sequences (https://jaspar.genereg.net/) [25].

## 3. Results

### 3.1. Excellent Sample Data Quality with High Confidence

**Figure 1:**
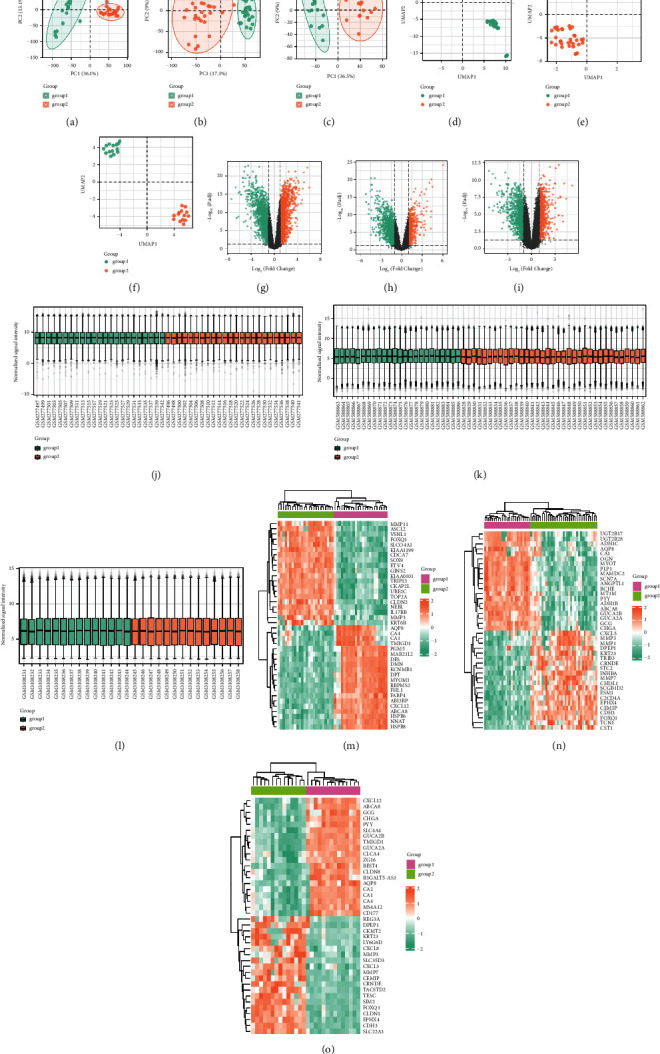
Obtain differential genes from GEO dataset. (a–c) PCA plot of GSE10972, GSE23878, and GSE113513. (d–f) UMAP plot of GSE10972, GSE23878, and GSE113513. (j–l) Sample normalization plot. (m–o) Heat map of GSE10972, GSE23878, and GSE113513.

After downloading the expression profile data, it is essential to assess the quality of the information [26]. Principal component analysis (PCA) maps show the features of high-latitude data by extracting their feature vectors, which are then converted into low-dimensional data, and using two-dimensional maps. The distance between samples indicates the differences between them. The figure showed that the characteristics of samples in the same group are similar, and the differences in samples between different groups are significant (Figures 1(a)–1(c)). The Uniform Manifold Approximation and Projection (UMAP) map was also used to determine differences between samples in the dataset expression profile (Figures 1(d)–1(f)). The volcano plot is used for visualizing the results obtained after difference analysis. Each point in the volcano plot represents the difference multiple of one molecule and the converted corrected *p* value (Figures 1(g)–1(i)). A normalized box plot was used to examine the chip strengths of the molecules in each sample in the dataset (Figures 1(j)–1(l)). The first 40 genes with significant differences in each dataset were visualized in the form of heat maps (Figures 1(m)–1(o)). From the three datasets, there were 113 common downregulated genes and autophagy gene intersections, and 73 common upregulated genes and autophagy gene intersections (Supplementary Figure 1A–1B).

### 3.2. Screening Genes to Obtain the Key Gene Associated with Ferroptosis and Autophagy

**Figure 2:**
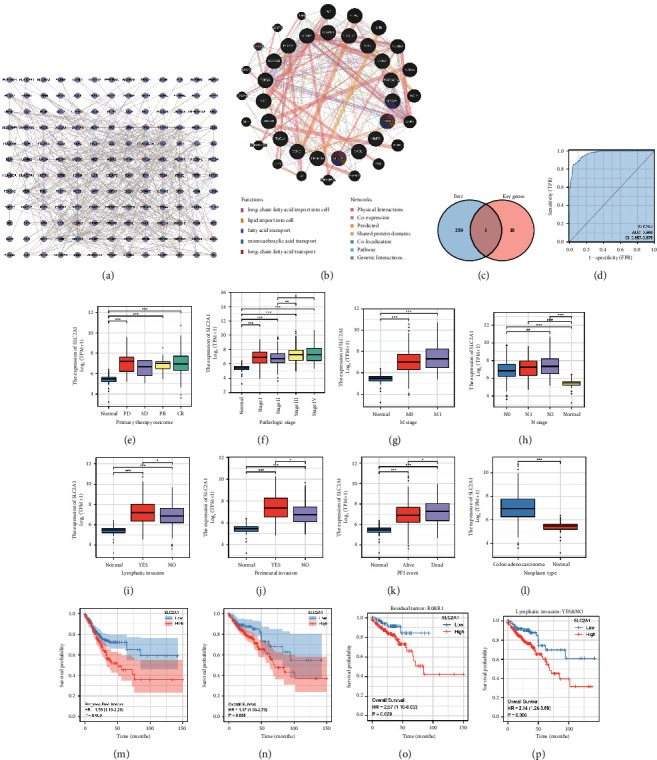
The excellent prognostic value of SLC2A1. (a, b) Protein interaction networks obtained from STRING and GeneMANIA. (c) The intersection of FerrDB genes and 19 key genes. (d) The ROC curve. (e–n) The strong positive correlation of SLC2A1 with clinical conditions such as primary therapy outcome and pathological stage. (o–r) OS and PFI showed that SLC2A1 has great prognostic significance for patients' survival, and different subgroups also confirmed the status of SLC2A1 as a key prognostic marker.

We crossed the differential genes analyzed from the three expression profiles with the autophagy genes obtained from GeneCards to obtain a gene set containing 186 genes. Then we input the gene set into STRING, and a complex and comprehensive protein-protein interaction network diagram was produced (Figure 2(a)). After the unconjugated proteins were removed, the protein interaction data were imported into Cytoscape to calculate the Maximal Clique Centrality (MCC) value, and 156 genes were found to be involved in this interaction network. We selected 55 genes with an MCC value greater than 10 and then used the clinical data from TCGA to explore their survival significance; from this, we obtained 19 genes with clinical significance. Next, we intersected the ferroptosis gene set obtained from FerrDb with the 19 genes and finally obtained the SLC2A1 gene (Figures 2(b)-2(c)). In previous studies, researchers used all-trans retinoic acid (ATRA) to treat different lung cancer cells and found that the most significant reduction in the transcription level of SLC2A1 was found in SLC2A1 and the genes in the same family, thus excluding other gene families [5]. Therefore, we determined that the SLC2A1 gene not only plays an important role in ferroptosis but also plays an indispensable role in autophagy in colon cancer. In order to explore the clinical relevance of SLC2A1 expression in colon cancer, clinical data from TCGA were downloaded for analysis and SLC2A1 was found to have high predictive power for the variable SLC2A1 in predicting the outcomes of Normal and Tumor (AUC = 0.968, CI = 0.957–0.979, Figure 2(d)). In the main treatment outcome, PD, PR, and CR were all highly correlated with the expression level of SLC2A1 and all of them were statistically significant (Figure 2(e)). Regarding pathological stages, the expression of SLC2A1 increased as the stages progressed (Figure 2(f)). In stages M and N of the tumor, the expression level of SLC2A1 increased slowly as the tumor progressed, and it could be seen that the high expression of SLC2A1 seriously affected the deterioration of the tumor (Figures 2(i)-2(j)). Moreover, SLC2A1 expression levels in patients with lymphoid invasion were higher than those in patients without lymphoid invasion, and SLC2A1 expression levels in patients with perineural invasion were higher than those in patients without perineural invasion (Figures 2(k)-2(l)). More importantly, in terms of PFI, SLC2A1 expression levels were higher in deceased patients than in surviving ones (Figures 2(m)-2(n)). The KM prognostic curve also illustrates the excellent prognostic value of SLC2A1 in both PFI and OS prognosis types (Figures 2(o)-2(p)). In addition, both high SLC2A1 expression groups had poor prognostic outcomes in the presence of residual tumor and lymphoid invasion (Figures 2(q)-2(r)). We also performed univariate and multivariate COX regression using TCGA data and plotted the baseline data table (Supplementary-Tables 1-2, Supplementary Figure 1C).

### 3.3. Transcriptomics and Proteomics Confirmed the High Expression Level of SCL2A1 in Colon Cancer

**Figure 3:**
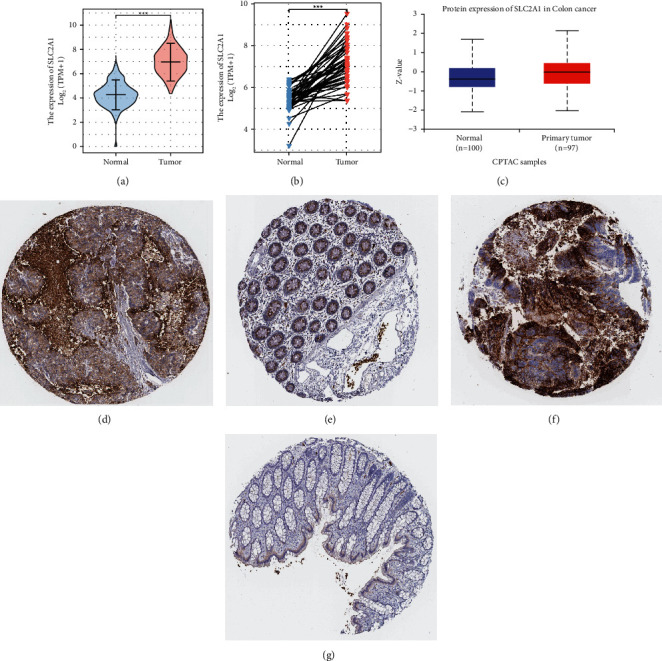
The verification of SLC2A1 expression by transcriptomics and proteomics. (a) Unpaired samples. (b) Paired samples. (c) SLC2A1 protein expression in the UALCAN database. (d–g) Immunohistochemistry in the HPA database demonstrated the difference in protein abundance of SLC2A1 in tumor and constituent tissues.

From the unpaired sample data from TCGA, the Mann Whitney *U* test (Wilcoxon rank-sum test) showed that Tumor was higher than Normal, and the median difference between the two groups was 2.701 (2.531–2.867), with a statistically significant difference (*p* < 0.001). The paired sample *t*-test showed that Tumor was above the average level of Normal, and the difference between the two groups was 1.774 (1.417–2.132), with a statistically significant difference (*t* = 10.026, *p* < 0.001). The result was a worse prognosis in the high group (Figures 3(a)-3(b)). In addition, we explored the protein expression of SLC2A1 in the HPA and CPTAC databases, and we found that the protein abundance of SLC2A1 in tumors was much higher than that in normal tissues (Figure 3(c)–3(g)).

### 3.4. Multidatabase Joint Exploration of the Methylation Level of SLC2A1

**Figure 4:**
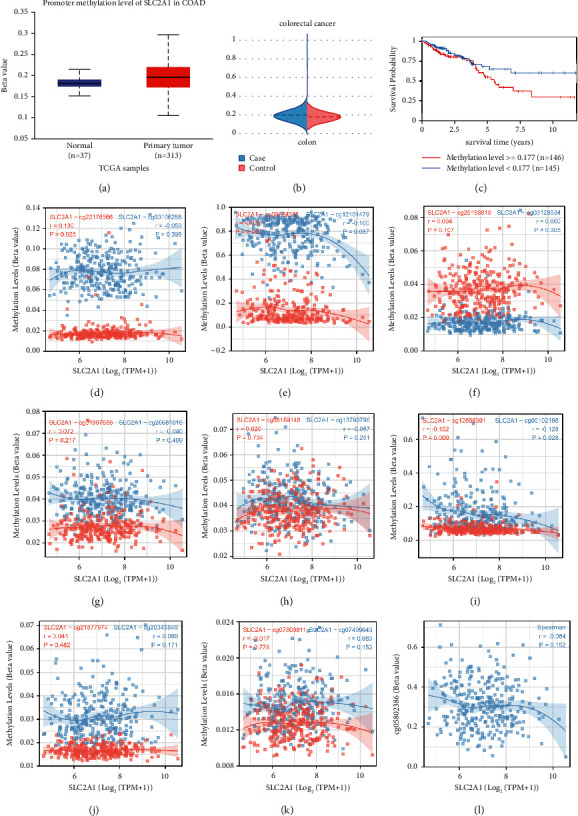
The DNA methylation of SLC2A1 in COAD. (a) Promoter methylation of SLC2A1 in UALCAN. (b, c) Methylation status and survival curve of SLC2A1 in EWAS. (d–l) Statistics for correlation between methylation status and expression status of SLC2A1 methylation probe in TCGA data.

N6-methyladenosine (m6A) is a post-transcriptional methylation modification that is widely distributed on the adenosine bases of RNA transcripts. This modifier is involved in the degradation of RNA transcripts, subcellular localization, and the regulation of splicing and local variations. In mammalian transcriptomes, only a small fraction of this motif is indeed modified, determining the other sequential and structural features of the m6A modification site. Results from both the UALCAN and EWAS databases revealed higher methylation levels of SLC2A1 in tumors and worse case outcomes in the hypermethylated group (Figures 4(a)–4(c)). After exploring the correlation between the methylation levels of SLC2A1 methylation probes and the transcription level of SLC2A1 in TCGA, we found that a total of five probes were correlated with the transcription level, with probes cg09824328, cg12656391, and cg00102166 being negatively correlated, and probe cg22176566 being positively correlated (Figures 4(d)–4(l)). After the sequence of SLC2A1 was downloaded from NCBI and the methylation sites of m6a were predicted using the SRAMP website, we found that there were a large number of methylation sites on the RNA of SLC2A1, and a total of 117 sites were obtained, including 16 sites with very high reliability. We also showed the RNA secondary structure of these 16 sites (Supplementary Table 3, Supplementary Figure 2). In MEXPRESS, we found that the previously mentioned probes exhibiting a negative correlation were validated against SLC2A1 expression levels. In addition, the expression level of SLC2A1 has a certain correlation with sample type, tumor stage, venous invasion, and lymphatic invasion. The heatmap also indicated that cg00102166 and cg12656391 showed obvious hypomethylation. At the same time, the methylation status of four methylation probes had considerable prognostic significance for survival. The group with low methylation of cg04287330 and cg13790786 had significantly poor prognostic outcomes, while the group with high methylation of cg20294984 and cg22025263 had even worse prognostic outcomes (Supplementary Figure 3).

### 3.5. Transcription Level of SLC2A1 Is Associated with Immune Infiltration in Tumors

**Figure 5:**
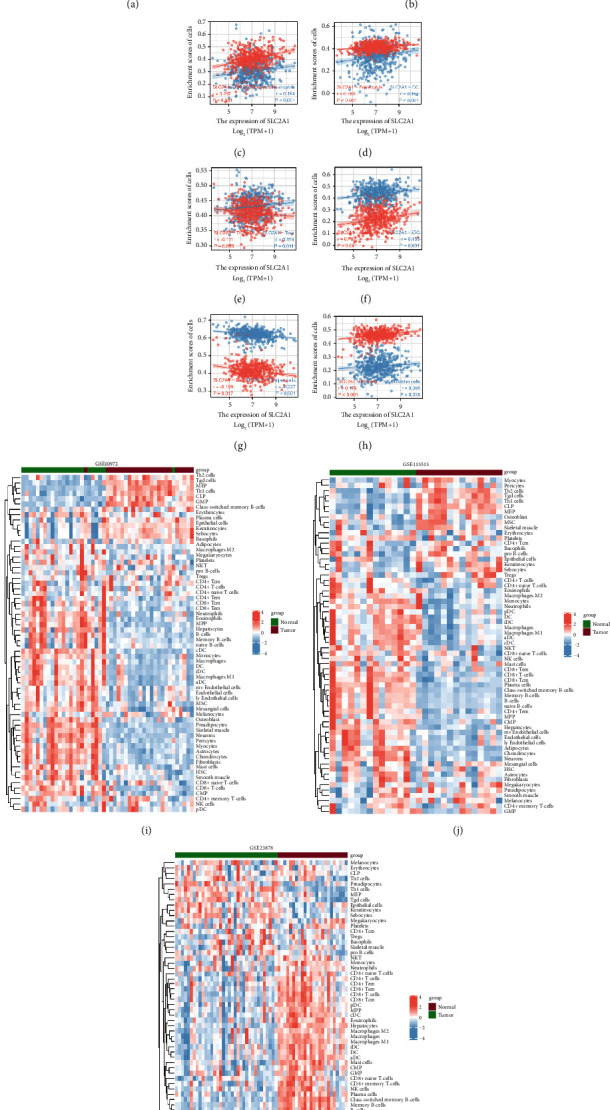
The immune infiltration of COAD and SLC2A1 was investigated according to the combined data of TCGA and GEO. (a–h) Correlation between the expression level of SLC2A1 in the TCGA database and the infiltration of different immune cells. (i–k) Immunoinfiltration analysis in xCell using expression profiles from three GEO datasets.

In order to explore the relationship between the transcription level of SLC2A1 and immune infiltration, we downloaded the RNAseq data from TCGA to study the infiltration level of multiple immune cells, including aDCs [activated dendritic cells], B cells, CD8 T cells, cytotoxic cells, DCs, eosinophils, iDCs [immature DCs], macrophages, mast cells, neutrophils, NK CD56 bright cells, NK CD56dim cells, NK cells, pDCs [plasmacytoid DCs], T cells, T helper cells, Tcm [T central memory] cells, Tem [T effector memory] cells, Tfh [T follicular helper] cells, Tgd [T gamma delta] cells, Th1 cells, Th17 cells, Th2 cells, and Treg cells. In the data of TCGA, we found that the expression level of SLC2A1 was correlated with some of these immune cells, and there was a positive correlation with NK CD56 bright cells, eosinophils, mast cells, NK cells, neutrophils, DCs, iDCs, Tem cells, and NK CD56dim cells. However, it was negatively correlated with Tcm, Th2, and helper cells (Figure 5(a)). The association of the high transcription level of SLC2A1 with the infiltration of a number of immune cells includes DCs, eosinophils, iDCs, mast cells, neutrophils, NK CD56bright cells, NK cells, and T helper cells (Figure 5(b)). At the same time, the correlation between the infiltration fraction of different immune cells and the transcription level of SLC2A1 can also be observed from the scatter diagram (Figures 5(c)–5(h)). We also performed a correlation analysis (Supplementary Table 4) for the key marker of each immune cell.

In order to systematically identify the cell states and cellular communities (ecotypes) in colon cancer, we used the EcoTyper tool to perform the analysis, inputting data from GSE10972, GSE23878, and GSE113513, which meet the stringent data requirements of CIBERSORTx, entered for analysis (Supplementary Figure 4). Tissue is a complex environment composed of numerous cell types. Understanding cellular heterogeneity in the tumor microenvironment is an emerging field of research in cancer. We used xCell to explore the tumor microenvironment in COAD using three datasets from GEO. The three heat maps reveal some interesting observations. Among the 64 types of immune cells, a small number of them were found to have a low infiltration fraction in normal tissue but a significantly increased infiltration fraction in tumor tissue. However, most types of immune cells revealed a high infiltration fraction in normal tissue but a significantly decreased infiltration fraction in tumor tissue, a phenomenon similar to “depletion” (Figures 5(i)–5(k)), such as typical T-cell depletion. Various T-cell subtypes are such as CD4+ Tcm; CD4+ T cells; and CD4+ naïve T cells such as CD8+Tcm. In addition, we found that M1 and M2 macrophages, various B cell subtypes, and different subtypes of endothelial cells all share the same cell state as T cells. After initial T cells are activated by antigens, costimulation and inflammation, they proliferate exponentially and differentiate into effector T cells and memory T cells. At the same time, we can also observe that the total immune infiltration of the tumor tissue and the sum of the scores of all types of immune cells are much lower than those of the normal tissue, indicating that the patient's immune response was decreased, and it even promoted the immune escape of the tumor cells (Supplementary Figure 5).

## 4. Discussion

Colon cancer is the third most common malignant tumor in the world. Both ferroptosis and autophagy are thought to be involved in the development of colon cancer, but the relationship between them has not been revealed. To explore the mechanisms underlying the association between ferroptosis and autophagy in colon cancer, we downloaded three high-quality colon cancer datasets from the GEO database and analyzed differentially expressed genes. We then intersected the differentially expressed genes with autophagy-related genes to obtain a gene set containing 186 genes. A protein-protein interaction network was constructed for this gene set, and genes with low correlation were eliminated. At the same time, survival analysis was performed using TCGA data, with 19 genes with prognostic significance being considered as key genes. Subsequently, we found the intersection between ferroptosis-related genes and key genes to obtain SLC2A1 as a potential marker gene. The expression level of SLC2A1 itself is associated with the prognosis of colon cancer patients. SLC2A1 has a large number of methylation sites, and the methylation of SLC2A1 is related to T cell exhaustion and immunosuppression in tumor tissue.

SLC2A1 encodes the major glucose transporter in the mammalian blood-brain barrier. The encoded protein is mainly present in the cell membrane and cell surface and promotes the glucose transporter responsible for constitutive or basal glucose uptake [27–30]. SLC2A1 can affect functions such as carbohydrate homeostasis and carbohydrate kinase activity. The increase in SLC2A1 expression may stimulate cellular glycolysis, and our analysis also suggests that the expression of SLC2A1 may contribute to the Warburg effect and promote tumor cell metastasis (Supplementary-Figure 6A). Previous studies have shown that high transcription levels of SLC2A1 may lead to lymphoid and perineural invasion [31], suggesting that SLC2A1 plays a conserved function in a variety of malignancies.

By further investigating the mechanism by which SLC2A1 affects prognosis, we learned that SLC2A1 is closely related to HK family proteins [32]; therefore, we explored the epigenetic modification of SLC2A1, which has been shown in past studies to be hypermethylated with high levels of transcription, and the protein level is high in colon cancer [33]. We predicted the methylation sites of SLC2A1 and found that the SLC2A1 sequence contained a large number of m6a modification sites (with 16 very high-confidence sites); methylation sites were also revealed in the RNA secondary structure. The correlation between SLC2A1 and the expression levels of several methylase and autophagy-related proteins was also explored, and SLC2A1 was found to be associated with these proteins (Supplementary Figure 6B). At the same time, we studied the correlation between the expression levels of several probes, which also proved that the overall DNA methylation level of SLC2A1 and the methylation levels of these probes were significantly associated with prognosis.

According to the literature, when SLC2A1 is significantly activated and transcribed by the DNA methylation modifier, lymphoid-specific helicase will significantly inhibit ferroptosis, which also leads to significantly reduced lipid peroxidation and ROS [34]. However, after the deacetylase Sirtuin 2 (SIRT2) is inactivated by ROS, acetylated Forkhead Box O1 (FoxO1-Ac) accumulates in the cytoplasm and complexes with Autophagy Related 7 (ATG7), which can stimulate autophagy. This autophagy-inducing mechanism keeps FoxO1 inactive as a transcription factor in the cytoplasm but still inhibits the growth of tumor xenografts in nude mice [35, 36]. Our results suggest that methylation of SLC2A1 is associated with immunosuppression in colon cancer. Chronically high expression of SLC2A1 may lead to prolonged antigen exposure of immune cells and inflammation. Effector T cells are continuously stimulated, transforming them into exhausted T cells; T cell exhaustion is one of the major factors in immune dysfunction in cancer patients. This leads to immune escape and tumor progression. These results suggest that the expression and hypermethylation of SLC2A1 affects the processes of ferroptosis and autophagy, which in turn affects tumor immunity.

Our study substantiates that the ferroptosis and autophagy-co-associated gene SLC2A1 is involved in tumor immune regulation and tumor prognosis in colon cancer. Hypermethylation of SLC2A1 is associated with T cell exhaustion and suppression of the immune microenvironment in colon cancer, a process which is most likely mediated by ferroptosis and autophagy. However, further research is required to determine the specific biological mechanism for this. The limitation of this study is that the data were obtained using exclusively bioinformatic methods; thus, there is a lack of experimental data. In conclusion, our study demonstrates that SLC2A1 is involved in the regulation of ferroptosis and autophagy in colon cancer, which in turn regulates tumor immunity and affects tumor progression. Our results also indicate that SLC2A1 may become an effective therapeutic target for colon cancer in the future.

## Data Availability

The data of this study are available from the public databases.

## Supplementary Materials

Supplementary Materials Supplementary Figure 1. Intersection of differential genes and autophagy genes. A: there were 113 intersecting genes of common downregulating genes and autophagy genes. B: there were 73 intersecting genes of coupregulated genes and autophagy genes. C: forest map is shown. Supplementary Figure 2. The m6a site on the SLC2A1 sequence and its secondary RNA structure are predicted. A: distribution map of m6a prediction sites. B–Q: secondary RNA structure with very high-confidence methylation sites. Supplementary Figure 3. Comprehensive exploration of the methylation probes of SLC2A1. A: heat map of SLC2A1 in MEXPRESS is illustrated. B: heat map of SLC2A1 in MethSurv is shown. C–F: methylation probes of clinical significance in SLC2A1 are depicted. Supplementary Figure 4. Analysis of GEO data using EcoTyper. A: the row of the heat map corresponds to cell state, the column corresponds to sample, and the colour corresponds to cell state abundance. The colour bar on the left represents the state of each cell, the cancer ecotype it forms, its cell type, and its ID. The top colour bar indicates the cancer ecotype with the highest abundance in each sample. B–M: a heat map is shown on the left, which depicts the cell type-specific expression of cell state characteristics of genes in TCGA cancer samples for reference. The right side shows the heat map of gene expression of cell state characteristics in GEO data. N: classification diagram of cell interaction state is shown. Supplementary Figure 5. Stacked histogram of immune infiltration scores. A-B: distribution of immune scores of GSE23878 and GSE113513 in CIBERSORTx are illustrated. C–E: immune infiltration scores of GSE 10972, GSE23878, and GSE113513 in xCell are shown. Supplementary Figure 6. Biological functions affected by SLC2A1 and correlation with specific proteins. A: Effects of SLC2A1 can be seen in GeneMANIA. B: correlation between SLC2A1 expression levels and methylase, autophagy and ferroptosis proteins in COAD is shown. Supplementary Table 1. Univariate/multivariate COX regression. Supplementary Table 2. Baseline data sheet of SLC2A1. Supplementary Table 3. Prediction of SLC2A1 methylation sites in SRAMP. Supplementary Table 4. Correlation between SLC2A1 and key marker of immune cells.
