# Supplementary material for: Methylation Drives SLC2A1 Transcription and Ferroptosis Process Decreasing Autophagy Pressure in Colon Cancer
Source: J Oncol. 2022 Aug 27;2022:9077424. doi: 10.1155/2022/9077424 (PMC9440784; doi:10.1155/2022/9077424)
Supplement: Supplementary Materials — Supplementary Figure 1. Intersection of differential genes and autophagy genes. A: there were 113 intersecting genes of common downregulating genes and autophagy genes. B: there were 73 intersecting genes of coupregulated genes and autophagy genes. C: forest map is shown. Supplementary Figure 2. The m6a site on the SLC2A1 sequence and its secondary RNA structure are predicted. A: distribution map of m6a prediction sites. B–Q: secondary RNA structure with very high-confidence methylation sites. Supplementary Figure 3. Comprehensive exploration of the methylation probes of SLC2A1. A: heat map of SLC2A1 in MEXPRESS is illustrated. B: heat map of SLC2A1 in MethSurv is shown. C–F: methylation probes of clinical significance in SLC2A1 are depicted. Supplementary Figure 4. Analysis of GEO data using EcoTyper. A: the row of the heat map corresponds to cell state, the column corresponds to sample, and the colour corresponds to cell state abundance. The colour bar on the left represents the state of each cell, the cancer ecotype it forms, its cell type, and its ID. The top colour bar indicates the cancer ecotype with the highest abundance in each sample. B–M: a heat map is shown on the left, which depicts the cell type-specific expression of cell state characteristics of genes in TCGA cancer samples for reference. The right side shows the heat map of gene expression of cell state characteristics in GEO data. N: classification diagram of cell interaction state is shown. Supplementary Figure 5. Stacked histogram of immune infiltration scores. A-B: distribution of immune scores of GSE23878 and GSE113513 in CIBERSORTx are illustrated. C–E: immune infiltration scores of GSE 10972, GSE23878, and GSE113513 in xCell are shown. Supplementary Figure 6. Biological functions affected by SLC2A1 and correlation with specific proteins. A: Effects of SLC2A1 can be seen in GeneMANIA. B: correlation between SLC2A1 expression levels and methylase, autophagy and ferroptosis proteins in COAD is s [file 9077424.f1.zip › sup-figures and sup-tables/Suppl-table3.docx]

Suppl-table3 Prediction of SLC2A1 methylation sites in SRAMP.

|  | Position | Sequence context | Structural context | Score(binary) | Score(knn) | Score(spectrum) | Score(combined) | Decision |
| --- | --- | --- | --- | --- | --- | --- | --- | --- |
| 1 | 125 | GAGTC GCAGT GGGAG | PBPPP PPMPI PPPPP | 0.537 | 0.704 | 0.513 | 0.536 | m6A site (Low confidence) |
|  |  | TCCCC GGACC GGAGC | IPPPI PPMPP PPMMM |  |  |  |  |  |
|  |  | ACGAG CCTGA GCGGG | MMPII PPPPI PPPPP |  |  |  |  |  |
| 2 | 282 | CCCGC CAGGA ACAAA | PPPPP PPPPI IPMPP | 0.569 | 0.779 | 0.513 | 0.557 | m6A site (Low confidence) |
|  |  | GGCGC AGACT CCTGC | PPPPP PPHHH HPPPP |  |  |  |  |  |
|  |  | CCGCC TTACG CCGGC | MMPPP PPMMM PPPPP |  |  |  |  |  |
| 3 | 542 | CGCGG CCCTG CTGGG | PBPPP PPPHH HHHPP | 0.613 | 0.832 | 0.448 | 0.558 | m6A site (Moderate confidence) |
|  |  | GCTGG GGACA CAGGG | PPPPP PPPPM MMPPP |  |  |  |  |  |
|  |  | CCCGG GAGCG GCACG | PPPPP PBPPP PPMMP |  |  |  |  |  |
| 4 | 1096 | GGTTG GGGAA TGAGG | PPMPP PIIPP MPPPP | 0.7 | 0.718 | 0.509 | 0.625 | m6A site (High confidence) |
|  |  | GATGG GGACT CTGGG | PPPPP PPPPP PPPPP |  |  |  |  |  |
|  |  | GACCA ACAAC TCCCT | PPHHH HHHHH PPPPP |  |  |  |  |  |
| 5 | 1415 | CGGGG GTAGC ACTGA | PPPPP PPMMM MMMMM | 0.59 | 0.539 | 0.559 | 0.575 | m6A site (Moderate confidence) |
|  |  | ATTTT GAACT GCCCT | MMPPP PIPPP IIPPP |  |  |  |  |  |
|  |  | TCCTA CTGGC TGTGG | PIPPP PPPPP PPBPP |  |  |  |  |  |
| 6 | 1453 | GCTGT GGAGG TGTCC | PPPPB PPPPP PIPPI | 0.619 | 0.8 | 0.544 | 0.598 | m6A site (Moderate confidence) |
|  |  | CGCTG AGACT GAGCG | PPPPP PPBPP MMPIP |  |  |  |  |  |
|  |  | GGGTG GCGCT GGGCC | PPPPP PMMMM PPPPP |  |  |  |  |  |
| 7 | 3017 | TTAGC TGAAT AGGAA | IPPPI IPPPP PPPPP | 0.599 | 0.524 | 0.453 | 0.537 | m6A site (Low confidence) |
|  |  | GTAGG GGACA GATGG | PPMPP PPPPP PBPPP |  |  |  |  |  |
|  |  | AATCA CCTTG CCTAC | PPMMM MMPII PPPII |  |  |  |  |  |
| 8 | 3126 | CTCAC CCCAC CCTTC | PPPPP PPMPP PPPMM | 0.664 | 0.557 | 0.561 | 0.617 | m6A site (High confidence) |
|  |  | GTGAA GAACT TGTAA | MMMMM MMMMP PPPPI |  |  |  |  |  |
|  |  | CTAGC CTTGC CTATG | IIPPP PPPPP PPMMM |  |  |  |  |  |
| 9 | 4110 | CCTCA TGGTC CAATA | PPHHH HPPPP MMMPP | 0.628 | 0.654 | 0.607 | 0.621 | m6A site (High confidence) |
|  |  | AAAAA TGACT TTGCC | PPPPP BPBPM MMPPI |  |  |  |  |  |
|  |  | AAGAT CACAC ACTGA | PPPPM MMMMM MMMMM |  |  |  |  |  |
| 10 | 4148 | ACACT GAGCC TCCCA | PIIPP PPIPP PPPPM | 0.46 | 0.406 | 0.67 | 0.541 | m6A site (Low confidence) |
|  |  | CCCCA AGACA TGCTG | MPPPP PMMMM MMMPP |  |  |  |  |  |
|  |  | CCAGC CTTTC CAGGA | PPMMP PPHHH HPPPM |  |  |  |  |  |
| 11 | 4173 | TGCTG CCAGC CTTTC | MMMPP PPMMP PPHHH | 0.465 | 0.68 | 0.663 | 0.555 | m6A site (Low confidence) |
|  |  | CAGGA TGACA TTAGA | HPPPM MPPPM MMMPP |  |  |  |  |  |
|  |  | GGGTG GAAGG AACCC | PPMPP PPPPP IIPPP |  |  |  |  |  |
| 12 | 4729 | AGCAG GAATC CCCAG | BPPPP PBBPP PHHHH | 0.713 | 0.838 | 0.589 | 0.669 | m6A site (High confidence) |
|  |  | ACACA GGACT TGCCA | HHHHH PPPPP PPPPP |  |  |  |  |  |
|  |  | TGCCA TAGGA GCCTG | PMMMM MMPPP PPPMM |  |  |  |  |  |
| 13 | 4784 | TAGAC GTAAC TTGAC | IPPHH HHHHP PIIIP | 0.469 | 0.506 | 0.62 | 0.531 | m6A site (Low confidence) |
|  |  | AACTG AAACT CTACT | PPPPM MMMPP PPPPP |  |  |  |  |  |
|  |  | GTGAA TGGAA ACAAA | PIPPP BPPPP PPMMM |  |  |  |  |  |
| 14 | 4887 | GTTTC CTTCT CAGTA | PPPPP PPPPI PPPPB | 0.644 | 0.073 | 0.576 | 0.588 | m6A site (Moderate confidence) |
|  |  | AATAG AGACT TGGCT | BBBPP PPMMM MPPPP |  |  |  |  |  |
|  |  | ACCCC CAGTG GATGT | BPPMM MMPPP PPIPP |  |  |  |  |  |
| 15 | 4916 | TACCC CCAGT GGATG | PBPPM MMMPP PPPIP | 0.597 | 0.519 | 0.507 | 0.557 | m6A site (Low confidence) |
|  |  | TGGTG AGACT GGCCC | PPPPP PPPPI PPPII |  |  |  |  |  |
|  |  | CGTGC CCTAA ATTAG | IPPPB BBBBB BBBBP |  |  |  |  |  |
| 16 | 5212 | TCCAG CTCCC TGCTT | PPPPP PPPPP PPPPP | 0.652 | 0.548 | 0.44 | 0.562 | m6A site (Moderate confidence) |
|  |  | CCCCA AGACT GGCAA | IIPPP HHHHP PPIIP |  |  |  |  |  |
|  |  | AGCTG GGGAG GCCTG | PPPBP PPPPP BBPPP |  |  |  |  |  |
| 17 | 5491 | CCTCT CTCCA CGGGA | PPPPP PPHHH HHPPP | 0.666 | 0.659 | 0.662 | 0.664 | m6A site (High confidence) |
|  |  | GGGGT GGACT GGAGC | PPPPP PPIPP PPIPP |  |  |  |  |  |
|  |  | CTGAG GCTCT GTCTT | PPPMP PPPHH HHHHH |  |  |  |  |  |
| 18 | 5548 | CCTGG CAGTA GCTGA | PPPPP PMPPP PPPPM | 0.426 | 0.518 | 0.672 | 0.529 | m6A site (Low confidence) |
|  |  | TGCAT AAACA TTAGG | PPPPB PPPPH HHHHH |  |  |  |  |  |
|  |  | GTTTT GCAGT AACTG | PPPPP PPPMM MMMPP |  |  |  |  |  |
| 19 | 5567 | TAAAC ATTAG GGTTT | BPPPP HHHHH HPPPP | 0.459 | 0.421 | 0.67 | 0.541 | m6A site (Low confidence) |
|  |  | TGCAG TAACT GAGCA | PPPPM MMMMP PPPPM |  |  |  |  |  |
|  |  | GGGCT CCCAG GCCTT | PPPPP PPPPI PPIIP |  |  |  |  |  |
| 20 | 5639 | TCCTC TCTTT GTGAC | PBBBB PPPPP MPPBP | 0.537 | 0.755 | 0.66 | 0.597 | m6A site (Moderate confidence) |
|  |  | CACAC TGACT TGCCT | PMMMP PPIPP PPPPP |  |  |  |  |  |
|  |  | CCCTG AGGCT TCTGC | PPPII PPIPP IPPPP |  |  |  |  |  |
| 21 | 5733 | TGGCT ACTGA GGCCT | MPPPP PIIPP PPPII | 0.593 | 0.519 | 0.64 | 0.608 | m6A site (High confidence) |
|  |  | CTGCT GGACA GGAAG | IIPPI PPPPH HHHHP |  |  |  |  |  |
|  |  | TCTGC CTATG TCTCC | PPPPP IIIIP PPPPI |  |  |  |  |  |
| 22 | 6053 | CCTCT GCTGG AGGAA | PPIPP PPPPP PPPII | 0.73 | 0.776 | 0.475 | 0.63 | m6A site (High confidence) |
|  |  | GAGGT GGACT CTCTT | IIIPP PIIPP PMMPP |  |  |  |  |  |
|  |  | GGGCT GGAGG GCCCA | PPPPP PPIPP PPIPP |  |  |  |  |  |
| 23 | 6145 | GCTGT GGCAG TTCTT | PBPPP PPPIP PPIPP | 0.623 | 0.644 | 0.397 | 0.533 | m6A site (Low confidence) |
|  |  | CTTGA GGACT TCCTG | PPPMM PPPPP PPMMP |  |  |  |  |  |
|  |  | CCCCT AGGTG GGGGC | PPPPP HHHHP PPPPP |  |  |  |  |  |
| 24 | 7884 | TAACT GAAGC TTAGA | IIIII IIIPP PIPPB | 0.632 | 0.425 | 0.544 | 0.586 | m6A site (Moderate confidence) |
|  |  | GGAAG AGACT TTTTT | PPPPP PMMMM MMMMM |  |  |  |  |  |
|  |  | TTGAG ACGGA GTTTC | MPPPP IPPPP PIIPP |  |  |  |  |  |
| 25 | 8187 | CACCA TGCCC AGCCA | PMMMP PPPMM MPPPP | 0.817 | 0.529 | 0.442 | 0.652 | m6A site (High confidence) |
|  |  | GGAAA GGACT TTTTT | PPPII PPPPP PMMMM |  |  |  |  |  |
|  |  | AAGGT CACAC AAGTG | PPPPP BPPPP PIPPP |  |  |  |  |  |
| 26 | 8221 | TCACA CAAGT GGAGG | PBPPP PPIPP PPIIP | 0.661 | 0.599 | 0.453 | 0.575 | m6A site (Moderate confidence) |
|  |  | AGCTG GGACT TAAAT | PPIPP PPIII IIPPP |  |  |  |  |  |
|  |  | GTGTC TGCTG AGAGT | PIIPP IPPPP PPPPB |  |  |  |  |  |
| 27 | 8954 | AGAAA CCTTG TTTCC | PPMMM PPPPP PPPPP | 0.589 | 0.5 | 0.548 | 0.568 | m6A site (Moderate confidence) |
|  |  | TTCAG GGACC AGTAC | BBBBP PPHHH HHHHP |  |  |  |  |  |
|  |  | CCGGA AACAA GGGGG | PPPPP PPPPP PPPPP |  |  |  |  |  |
| 28 | 8980 | GTACC CGGAA ACAAG | HHHPP PPPPP PPPPP | 0.573 | 0.576 | 0.551 | 0.564 | m6A site (Moderate confidence) |
|  |  | GGGGA GGACC TAAGC | PPPPP PPIII IIIPP |  |  |  |  |  |
|  |  | AGGAA AGCCA TAGAA | PPPPI IIIIP PPPPP |  |  |  |  |  |
| 29 | 10345 | CCCCC ACTCC TTTGA | PPPII IPPPP PIIPP | 0.707 | 0.749 | 0.554 | 0.648 | m6A site (High confidence) |
|  |  | GCCGT GGACT AAGCA | PPPPP PPPMM MMMMM |  |  |  |  |  |
|  |  | GTTGG AAAGC CCTGC | PPPPP MMMMP PPIPP |  |  |  |  |  |
| 30 | 10421 | TGTGG GATGC TGTGG | BPIPP PPPPP IIIPP | 0.617 | 0.647 | 0.566 | 0.598 | m6A site (Moderate confidence) |
|  |  | GAAGA GGACA AGATG | PMMMM PPPPI PPPPP |  |  |  |  |  |
|  |  | GAGCA ACATC TGGAG | HHHHH HPPPP PIIIP |  |  |  |  |  |
| 31 | 10459 | TCTGG AGTCT GCCAG | PPPII IPPPP MPPPP | 0.504 | 0.564 | 0.656 | 0.567 | m6A site (Moderate confidence) |
|  |  | CCCTG GGACC CTCCT | PMMMP PPPPP PPPPP |  |  |  |  |  |
|  |  | CTTGC CAGGA ATACC | PPPPP IIPPP MMMMM |  |  |  |  |  |
| 32 | 10584 | GGAGC GCAAT GCTGA | PPIIP IPPPP BPPPI | 0.474 | 0.575 | 0.648 | 0.548 | m6A site (Low confidence) |
|  |  | CACAC TGACA TGGGT | IIIII PPIPP PPPPM |  |  |  |  |  |
|  |  | CAAAA GGCCA CAGTG | MMMMM PPPPP PPIPP |  |  |  |  |  |
| 33 | 11475 | GCAGA TGGGT AATAA | PPPII PPPPM MMMMP | 0.719 | 0.777 | 0.566 | 0.661 | m6A site (High confidence) |
|  |  | GCTAG AGACT GCAGA | PPMMP PPPPP PPMMM |  |  |  |  |  |
|  |  | GGACA TAGCA AGAGC | MMMMM MMPPH HHHPP |  |  |  |  |  |
| 34 | 11485 | AATAA GCTAG AGACT | MMMMM PPHHH HHHHH | 0.69 | 0.509 | 0.534 | 0.619 | m6A site (High confidence) |
|  |  | GCAGA GGACA TAGCA | PPMPP PPBPP PIPII |  |  |  |  |  |
|  |  | AGAGC AAAGG CCTGG | PPPPP MMMMP PPPPP |  |  |  |  |  |
| 35 | 11577 | GATGG GCTGC TGGGG | PPPPP PPPPM PPPPP | 0.587 | 0.692 | 0.495 | 0.555 | m6A site (Low confidence) |
|  |  | ATCCC AGACT CTGGG | PPPIP PPPPP PPPPP |  |  |  |  |  |
|  |  | CTGTA CATTG CTCCC | PPPPP PIPPP PBPPP |  |  |  |  |  |
| 36 | 11724 | TAAAT ACACG TTGAG | MMMMM PPPIP PPPBP | 0.746 | 0.6 | 0.512 | 0.645 | m6A site (High confidence) |
|  |  | TGAAT GGACT TCATT | PPPII IPPPP PMMMM |  |  |  |  |  |
|  |  | GCCAT GTTCC AGTTC | PPPMM MMMPP PPPHH |  |  |  |  |  |
| 37 | 11903 | ACAGC AACCC TTTAA | MMMMM MMMPP HHHHH | 0.673 | 0.539 | 0.548 | 0.616 | m6A site (High confidence) |
|  |  | GGAAG GAACT TTCAT | PPMMP PPHHH HHHHP |  |  |  |  |  |
|  |  | CCCCG TTTTC TAGGT | PPMPP PPPPP PPPPP |  |  |  |  |  |
| 38 | 12475 | AAACT CCTTC CTTGG | PPHHH HHPPP PMMMP | 0.674 | 0.65 | 0.387 | 0.558 | m6A site (Moderate confidence) |
|  |  | TGTCA GGACT GGGTC | PPIIP PPPPP PPIPI |  |  |  |  |  |
|  |  | CAGTT TCATC CGGGG | IPPPI IIIIP PPPPP |  |  |  |  |  |
| 39 | 12742 | GCTCA GTGGG GCCCC | PBPPP PPPPP PIPPP | 0.499 | 0.75 | 0.691 | 0.588 | m6A site (Moderate confidence) |
|  |  | AGGGA TGACT TCAGA | PPPPP PMMMM MMMPP |  |  |  |  |  |
|  |  | GGCTC CAGAC CCCTG | PPPIP IPPPI PPPPP |  |  |  |  |  |
| 40 | 14104 | GGCTA AGGGG TCAGG | PPPPI IPPMP PPHHH | 0.673 | 0.582 | 0.369 | 0.547 | m6A site (Low confidence) |
|  |  | TAGAC AGACT AATGG | HHPPP MMMMM MMMPP |  |  |  |  |  |
|  |  | GAAGG AAGGA TGCAG | PPMPP PBPII PPPPP |  |  |  |  |  |
| 41 | 14235 | GGCTT TTGCA GTCGT | HPPPP PIIPM PPPPP | 0.536 | 0.399 | 0.57 | 0.543 | m6A site (Low confidence) |
|  |  | TGCCC TGACT GACGC | PHHHH HPPPB PPPMM |  |  |  |  |  |
|  |  | TTTCA GAGAA TGAAA | MMPPP PPPMM PPIPP |  |  |  |  |  |
| 42 | 15040 | GGGGA GAGGT GGTAC | PPPMP PIIPP PPPPP | 0.751 | 0.721 | 0.435 | 0.623 | m6A site (High confidence) |
|  |  | ATGGG GGACT TAGCC | PPPPP PPPBB BPPPI |  |  |  |  |  |
|  |  | TGCTG GAATT CACAG | PIPPP PPIPP IIIIP |  |  |  |  |  |
| 43 | 15427 | GGCCA AGTTT CCTGG | PPPPM MPPPP PPPPP | 0.687 | 0.444 | 0.405 | 0.562 | m6A site (Moderate confidence) |
|  |  | GAGAA AGACT GGTGT | PBPPM PPPMP PPIPP |  |  |  |  |  |
|  |  | GGTGC CAAGA GTCAG | IPPPP PMPPI PPPMP |  |  |  |  |  |
| 44 | 15673 | CTGTG CCATC GTTGG | IIPPP MMMMM MPPPP | 0.611 | 0.462 | 0.59 | 0.595 | m6A site (Moderate confidence) |
|  |  | CTTAT GGACA CCAGC | PPPII IPPPI PPPPP |  |  |  |  |  |
|  |  | CTGCT CTGTT GCACA | MMPPB PPPPP PIIPP |  |  |  |  |  |
| 45 | 16137 | ATATC ACCCA GCTAA | MMMMM MMPPP PPPII | 0.55 | 0.594 | 0.609 | 0.576 | m6A site (Moderate confidence) |
|  |  | GTCAG AGACA GAGCT | PPPHH HPPPI IPPPP |  |  |  |  |  |
|  |  | GGTAT CCAAC ACCTG | PPMMM MPPPP MMPPP |  |  |  |  |  |
| 46 | 16474 | GGGTA GGGGG TAGGA | IPPPP PPPPP IPPPP | 0.622 | 0.543 | 0.405 | 0.531 | m6A site (Low confidence) |
|  |  | GGCAG GGACA GGAAA | PPPMP PPPII PPIIP |  |  |  |  |  |
|  |  | GGAAA CTGAG CCCTT | PHHHH PPIII PPIIP |  |  |  |  |  |
| 47 | 16970 | CAGCT GCAGG GGAGG | PPPPI PPIPP IIPPP | 0.741 | 0.413 | 0.526 | 0.638 | m6A site (High confidence) |
|  |  | TGACA GGACT TTTCC | PHHHH HHPPP PMMPP |  |  |  |  |  |
|  |  | AAGTT TGCTG GGCAT | IIPPI IPPPP PPPPP |  |  |  |  |  |
| 48 | 17016 | GAGAC CGTGG GCCCA | PMPPP PIPPP PPPBB | 0.555 | 0.626 | 0.54 | 0.553 | m6A site (Low confidence) |
|  |  | TGCTT GGACC AGCCC | PPBPP PPBPP PPPPP |  |  |  |  |  |
|  |  | CAAGC CCTGC ATTTG | PPPBP PPPPI IPPPP |  |  |  |  |  |
| 49 | 17081 | TTGCC AAAGT GTGTG | PPIPP PPPPB PBPIP | 0.62 | 0.503 | 0.469 | 0.554 | m6A site (Low confidence) |
|  |  | CTTCA GAACT GGCTC | PPHHH HHHHH PPPIP |  |  |  |  |  |
|  |  | CCTTT GGGAA TCCTT | PPPPP PPIPP MMPPP |  |  |  |  |  |
| 50 | 17343 | GTGAG TTAAT GACAG | HHPPP BBBPP IPPPP | 0.662 | 0.712 | 0.381 | 0.552 | m6A site (Low confidence) |
|  |  | AACTA GGACT CTAAG | IIIPP PPPPP PPPPP |  |  |  |  |  |
|  |  | GCCAG GACAG AGGAG | IPPPP MMMMM MPPPP |  |  |  |  |  |
| 51 | 17626 | GGGGC CCAGC TAGGG | PPMPP PPPPP PPPMM | 0.768 | 0.838 | 0.522 | 0.673 | m6A site (Very high confidence) |
|  |  | AGCAT GGACT TCAGG | MMMMP PMMPP PHHHH |  |  |  |  |  |
|  |  | ATGAG GACAA TAGGG | HHPPP MMPPM MMMPP |  |  |  |  |  |
| 52 | 17640 | GAGCA TGGAC TTCAG | PPBPP PPPMP PPPII | 0.695 | 0.863 | 0.578 | 0.657 | m6A site (High confidence) |
|  |  | GATGA GGACA ATAGG | IIPPP PPMMM MPPPP |  |  |  |  |  |
|  |  | GACCC ACTGC CCAGG | IIPPP BPPII PPPIP |  |  |  |  |  |
| 53 | 17649 | CTTCA GGATG AGGAC | PPPPI IIIPP PPPMM | 0.542 | 0.695 | 0.606 | 0.575 | m6A site (Moderate confidence) |
|  |  | AATAG GGACC CACTG | MMPPP PIIPP PBPPI |  |  |  |  |  |
|  |  | CCCAG GGCAG ACGTG | IPPPI PMPPP PPPPP |  |  |  |  |  |
| 54 | 17677 | TGCCC AGGGC AGACG | PIIPP PIPMP PPPPP | 0.571 | 0.525 | 0.617 | 0.587 | m6A site (Moderate confidence) |
|  |  | TGATC AGACT TGCAT | PPPPP PIIPP PPPHH |  |  |  |  |  |
|  |  | TGTAG GGAAA TGACT | HPPPP PIIII PPPBP |  |  |  |  |  |
| 55 | 17697 | AGACT TGCAT TGTAG | PIIPP PPPHH HPPPP | 0.539 | 0.656 | 0.688 | 0.604 | m6A site (High confidence) |
|  |  | GGAAA TGACT CAGGC | PIIII PPPBP PBBPP |  |  |  |  |  |
|  |  | GTCTG GTATG GAGGG | PPPPP MPIPP PIPPP |  |  |  |  |  |
| 56 | 17724 | GGCGT CTGGT ATGGA | BPPPP PPPMP IPPPI | 0.726 | 0.545 | 0.687 | 0.701 | m6A site (Very high confidence) |
|  |  | GGGGC GGACT TAAGA | PPPPB PIIPP PPMMM |  |  |  |  |  |
|  |  | ATGTG AGACT AGAGA | MPPPP IIPPP PPIPP |  |  |  |  |  |
| 57 | 17739 | GGGGC GGACT TAAGA | PPPPB PIIPP PPMMM | 0.557 | 0.484 | 0.654 | 0.592 | m6A site (Moderate confidence) |
|  |  | ATGTG AGACT AGAGA | MPPPP IIPPP PPIPP |  |  |  |  |  |
|  |  | CGGGA AACCA TCAAG | PPPII PPPHH HHHHH |  |  |  |  |  |
| 58 | 17776 | CCATC AAGAA AGTTG | PHHHH HHHHH HPPPI | 0.737 | 0.686 | 0.46 | 0.624 | m6A site (High confidence) |
|  |  | CCGTT GGACT AGTGA | PPPPP IIIPP PPPII |  |  |  |  |  |
|  |  | CATAA AAGCA AACCC | PPPPM MMMMM MMMPP |  |  |  |  |  |
| 59 | 18523 | CATCG GGTAC CCCAG | PPPPP MMMMM PPPPP | 0.639 | 0.772 | 0.438 | 0.565 | m6A site (Moderate confidence) |
|  |  | GGGTT GAACT GGGCA | HHHHH HHHPP PPPMM |  |  |  |  |  |
|  |  | AGCCT CTCCA CATTG | MPPPP PPIII IIPPP |  |  |  |  |  |
| 60 | 19208 | GAGCC ACCAT GCCCA | PHHHH HHPPP PPPMM | 0.763 | 0.713 | 0.484 | 0.649 | m6A site (High confidence) |
|  |  | GCCAG GGACT GAGAG | PPPPP PPMPP IPPPI |  |  |  |  |  |
|  |  | ACTTC TGAAG ATGTA | IIIPP PPHHH HHHPP |  |  |  |  |  |
| 61 | 19216 | ATGCC CAGCC AGGGA | PPPPP MMPPP PPPPM | 0.716 | 0.543 | 0.549 | 0.64 | m6A site (High confidence) |
|  |  | CTGAG AGACT TCTGA | PPIPP PIIII PPPPH |  |  |  |  |  |
|  |  | AGATG TAGAC AAATC | HHHHH PPPPI IIIPP |  |  |  |  |  |
| 62 | 19247 | GATGT AGACA AATCT | HHHHP PPPII IIPPP | 0.602 | 0.666 | 0.7 | 0.644 | m6A site (High confidence) |
|  |  | GAGAT GAACT TCTGT | IPPMM MMMMP PPPPP |  |  |  |  |  |
|  |  | GATCC CCGGG GCCTC | PBPPP PPPPP PPMMP |  |  |  |  |  |
| 63 | 19384 | GAGGG CCCAG ATGAG | MMMMP PPPPP PPBBP | 0.482 | 0.623 | 0.681 | 0.569 | m6A site (Moderate confidence) |
|  |  | CCTGT AAACT GCATT | PPPPP IIIPP MMMMM |  |  |  |  |  |
|  |  | CATAG GACCA TGTAT | MMMMM PPPPP HHHHP |  |  |  |  |  |
| 64 | 19398 | GCCTG TAAAC TGCAT | PPPPP PIIIP PMMMM | 0.519 | 0.67 | 0.692 | 0.596 | m6A site (Moderate confidence) |
|  |  | TCATA GGACC ATGTA | MMMMM MPPPP PHHHH |  |  |  |  |  |
|  |  | TGGTC ACCTG TCCAA | PPPPP MPPPP PPPMM |  |  |  |  |  |
| 65 | 19477 | GCTGG AGATG TGAGT | IPPPP BBBBP PPIIP | 0.617 | 0.703 | 0.66 | 0.638 | m6A site (High confidence) |
|  |  | GGCCA GAACT GAGAA | PIPPP PBBBB PPPPI |  |  |  |  |  |
|  |  | CGCTG AAATC TAGCG | PPPPP HHHHH PPPPP |  |  |  |  |  |
| 66 | 19538 | CACCC AGACA CTTGG | PPPPP PPIII IPPPP | 0.523 | 0.408 | 0.714 | 0.594 | m6A site (Moderate confidence) |
|  |  | TCTGT TGACT TTTGT | PPPPP PPMMM PPPPP |  |  |  |  |  |
|  |  | ACTTT ACAAA CACTT | PHHHP PPPPP MMMMM |  |  |  |  |  |
| 67 | 19634 | ACATG TAACA TGTGA | PPPPP HHHPP PPPPM | 0.635 | 0.735 | 0.594 | 0.623 | m6A site (High confidence) |
|  |  | AGAGA GGACA GAAGG | MMPPP PIPPP PPPPM |  |  |  |  |  |
|  |  | ACCCA AGAAG AAGAA | MPPPP PPPPP MMMMM |  |  |  |  |  |
| 68 | 19642 | CATGT GAAGA GAGGA | PPPPP PMMMP PPPIP | 0.596 | 0.65 | 0.624 | 0.61 | m6A site (High confidence) |
|  |  | CAGAA GGACC CAAGA | PPPPP PMMPP PPPPP |  |  |  |  |  |
|  |  | AGAAG AAATG AAAGT | PPMMM MMMMM MMMMM |  |  |  |  |  |
| 69 | 19702 | TGTGT AGTAA CAGGG | PPPHH HHHHP PPPPP | 0.645 | 0.487 | 0.659 | 0.643 | m6A site (High confidence) |
|  |  | CAGAG AGACT CCTGT | PMMMP PPIPP PIPPP |  |  |  |  |  |
|  |  | GCCCG CGTAG GCTGA | PPPPI IPPPP PPHHH |  |  |  |  |  |
| 70 | 19783 | CCAAC CCTTT CATTC | PPPII IIIIP PBPPP | 0.713 | 0.659 | 0.584 | 0.659 | m6A site (High confidence) |
|  |  | AGCCA GGACT TCCTG | PPPII PPMPP PPPPP |  |  |  |  |  |
|  |  | AGCAT CCACG CTGTG | PPIII PPPBP PPPPP |  |  |  |  |  |
| 71 | 19822 | GCTGT GCCAG ATGCG | PPPPP PPPMM MMMPI | 0.48 | 0.676 | 0.668 | 0.565 | m6A site (Moderate confidence) |
|  |  | GTCCT AGACA GTAAG | IPPPP HHHHH HHHPP |  |  |  |  |  |
|  |  | GAGAG AGAAG TATAG | PPIIP MMMMM MMMMP |  |  |  |  |  |
| 72 | 20286 | GTTTG CCTAC TCTTG | PPIPP PPPPP PMMMM | 0.725 | 0.632 | 0.451 | 0.611 | m6A site (High confidence) |
|  |  | AGAAG AGACT TTGCC | MMMMM PPPPP PPPPP |  |  |  |  |  |
|  |  | CCACA AGTGG TGAGG | PPMMM MPPPP PPPPP |  |  |  |  |  |
| 73 | 20483 | TTCCC CTCTT CTAAA | PPPPP PPPHH HHHHH | 0.637 | 0.42 | 0.47 | 0.559 | m6A site (Moderate confidence) |
|  |  | TGAGG GAACT GAAGC | HPPPP PMMMP PPPPP |  |  |  |  |  |
|  |  | CAGAT GAGGT ATCTG | PMMMP PIPPP PPIIP |  |  |  |  |  |
| 74 | 20596 | GCCCT GCTGT GAGTG | MMMPP PPPIP PMPPP | 0.618 | 0.691 | 0.653 | 0.635 | m6A site (High confidence) |
|  |  | TGACA GGACA TTCCT | PPIPP PPHHH HHPPP |  |  |  |  |  |
|  |  | GCCAT GCATC CCCTC | PIPPP PPMPP PPPPP |  |  |  |  |  |
| 75 | 20632 | CATCC CCTCC TGGAG | PMPPP PPPPP HHHHP | 0.676 | 0.814 | 0.645 | 0.67 | m6A site (High confidence) |
|  |  | GAGGA GGACA GAGCA | PPPPB PPPPP IPPPP |  |  |  |  |  |
|  |  | GAAGA GGTGA GCCCA | PMMMM MPPPB PPHHH |  |  |  |  |  |
| 76 | 20679 | CACAG AGGGA GCAGC | PPPMM PPPPI PPPPM | 0.473 | 0.644 | 0.756 | 0.595 | m6A site (Moderate confidence) |
|  |  | TAAAT GAACC AGGGA | MMMMM MMMMP PPPHH |  |  |  |  |  |
|  |  | ATCCT GACCA TCATA | HHPPP PMMPP PPPII |  |  |  |  |  |
| 77 | 20741 | CTGCC TTTCC CCTCC | IIPPP PPIPP PPPPM | 0.646 | 0.839 | 0.747 | 0.696 | m6A site (Very high confidence) |
|  |  | CCAAA TGACT GAGCT | MPPHH HHHHP PMPPI |  |  |  |  |  |
|  |  | GCCAG TAGAG ATGAC | PPPPP MMMPP PIIPP |  |  |  |  |  |
| 78 | 20762 | GACTG AGCTG CCAGT | HHHPP MPPIP PPPPM | 0.555 | 0.75 | 0.763 | 0.648 | m6A site (High confidence) |
|  |  | AGAGA TGACT GACGG | MMPPP IIPPP PPBPP |  |  |  |  |  |
|  |  | CCACT GAGAA ACCAT | PPMMP PHHHH HHPPM |  |  |  |  |  |
| 79 | 20803 | CCATG AAGTC CTGGC | HPPMM MMMMM PPPPP | 0.693 | 0.565 | 0.788 | 0.724 | m6A site (Very high confidence) |
|  |  | CCAAG GGACA GTGTC | PPPPP PPPII IPIPI |  |  |  |  |  |
|  |  | AGGCA TGTCT GAGAA | PPPBP PPPPP HHHHH |  |  |  |  |  |
| 80 | 21020 | GCCCT GTGTC CCGTC | PPPPI PPIII PPHHH | 0.632 | 0.571 | 0.468 | 0.563 | m6A site (Moderate confidence) |
|  |  | AGGCT GAACT GGGCT | HPPII IIPPI PPPPP |  |  |  |  |  |
|  |  | TCGTG CTCTA GGCAG | PMMMM PPPPP PMPPP |  |  |  |  |  |
| 81 | 21358 | CCTTG ACCAT TGTGG | PPPPP MPPPP PHHHH | 0.661 | 0.668 | 0.423 | 0.566 | m6A site (Moderate confidence) |
|  |  | AAGAT GGACA CAGTC | HHPPP PPMMM MMPPP |  |  |  |  |  |
|  |  | CCAGA TGGGC ACCTA | PPPPM MPPPP IIIII |  |  |  |  |  |
| 82 | 23691 | CTAGG CTCAG TGAGG | PPIPI IPPMM PPPPP | 0.58 | 0.796 | 0.509 | 0.562 | m6A site (Moderate confidence) |
|  |  | CACCT GGACA GAGAT | IIIPP PIPPP PHHHH |  |  |  |  |  |
|  |  | CTGTA GTAGC CCCTC | PPPPI IPPPI IPPPP |  |  |  |  |  |
| 83 | 23753 | CACCA TGGCA GTCCC | PPPPP PPPBP PIPPI | 0.627 | 0.727 | 0.571 | 0.609 | m6A site (High confidence) |
|  |  | CCCAG GAACT AACCA | PPPMP PHHHH HHPPM |  |  |  |  |  |
|  |  | GAACC GCTCC TCCCT | MMMMM PPPPP PPPPI |  |  |  |  |  |
| 84 | 23801 | CAACA CGCTT GCCGC | PPIII IIPPP IIIII | 0.538 | 0.743 | 0.722 | 0.621 | m6A site (High confidence) |
|  |  | CACCC AGACT CCACA | PPPPP PPBPP HHHHP |  |  |  |  |  |
|  |  | GCTGG GTGAA GAGAC | PPPPP PPPII PPPII |  |  |  |  |  |
| 85 | 23822 | GACTC CACAG CTGGG | PBPPH HHHPP PPPPP | 0.739 | 0.612 | 0.667 | 0.704 | m6A site (Very high confidence) |
|  |  | TGAAG AGACT GGGCT | PPIIP PPIIP PPPII |  |  |  |  |  |
|  |  | GGGAA GGGAG CAGAG | PPPPI IPPPP PMPPP |  |  |  |  |  |
| 86 | 23886 | CCTGG CACAT TTGGT | PPPPP PPPPP IIPPP | 0.643 | 0.57 | 0.561 | 0.607 | m6A site (High confidence) |
|  |  | GTGCA GGACT CCGAC | PIPPM PPHHH PPMPP |  |  |  |  |  |
|  |  | TGCAT TCTGA GGGGT | PPPMM MMPPP PPPPB |  |  |  |  |  |
| 87 | 24633 | AGCGT CCAGG AGCCA | MMMPP PPPPP BPHHH | 0.635 | 0.805 | 0.487 | 0.584 | m6A site (Moderate confidence) |
|  |  | GCCCT GGACA AGGTT | HPPPP PPPPM MPPPP |  |  |  |  |  |
|  |  | GGGTC CCTTT GACTG | PIPPP PPPII IIPPP |  |  |  |  |  |
| 88 | 24924 | AGGGT TGGGG GAATA | PPPPI PPPPP PPPPM | 0.637 | 0.582 | 0.545 | 0.597 | m6A site (Moderate confidence) |
|  |  | CCTTA GAACT ATGGA | MPPPP PPPPM PMMPP |  |  |  |  |  |
|  |  | ATGCA GAAAT TTAGC | PIPPP PPMMM MMMMP |  |  |  |  |  |
| 89 | 24971 | TCAGG AGCCA GTCTG | IIPPI PPPPP PPPPP | 0.626 | 0.624 | 0.627 | 0.626 | m6A site (High confidence) |
|  |  | CACAG GGACA AACAC | PPPPP PPBPM MMMMM |  |  |  |  |  |
|  |  | CTCTC CTTGC CCCTG | PPPPP PHHHH HHHHP |  |  |  |  |  |
| 90 | 25144 | AGGGC AAGCG CCATT | PPPPP MMPPI PPIPP | 0.589 | 0.409 | 0.493 | 0.542 | m6A site (Low confidence) |
|  |  | TATCT GGACC GTTCC | PPPPI PPIII IIIII |  |  |  |  |  |
|  |  | CATAT CCACC CTCAT | IIIIP PPPPP IIIII |  |  |  |  |  |
| 91 | 25860 | GGAAA TAAGG GGTGG | MMMMM MMMMM MMMPP | 0.503 | 0.315 | 0.631 | 0.544 | m6A site (Low confidence) |
|  |  | AGCTA GGACC TGAAT | PPPPI PPPPP PPPIP |  |  |  |  |  |
|  |  | TTAGT TGTGT CCAGA | PPIPH HHHHH PIPPP |  |  |  |  |  |
| 92 | 25933 | CTGCA TGGCG TCCTT | PPIPP PPPPP PPPPM | 0.619 | 0.59 | 0.629 | 0.621 | m6A site (High confidence) |
|  |  | GGAAC GGACA AGCAG | PPPIP HHHHH HHHHP |  |  |  |  |  |
|  |  | ATCCT TAGGG CTTGC | IPPPM MPPPP PBBPP |  |  |  |  |  |
| 93 | 26014 | GGCCT CCATC AGATG | PPPPP PPPPP PBBBB | 0.503 | 0.759 | 0.65 | 0.574 | m6A site (Moderate confidence) |
|  |  | CCTGC TGACT TGGGA | BPPPP PPBBP PPPMM |  |  |  |  |  |
|  |  | CAGGT TGTGA CAGGG | MMMMM MMMMM MMPPP |  |  |  |  |  |
| 94 | 26021 | ATCAG ATGCC TGCTG | PPPPB BBBBP PPPPP | 0.606 | 0.757 | 0.683 | 0.644 | m6A site (High confidence) |
|  |  | ACTTG GGACA GGTTG | BBPPP PMMMM MMMMM |  |  |  |  |  |
|  |  | TGACA GGGCA GGGGA | MMMMM PPPPP PPIPI |  |  |  |  |  |
| 95 | 26135 | TGAAT AGGTG GGCAT | HHHPP PPIPP PPPIP | 0.62 | 0.55 | 0.446 | 0.547 | m6A site (Low confidence) |
|  |  | TTCTG GGACT GCAGG | PPPPP PIIPP MPPPI |  |  |  |  |  |
|  |  | CTTTG GTTTG GCAAG | PPPPP PPPII IIIII |  |  |  |  |  |
| 96 | 27351 | TACCT CTCTG TTCTA | PIIII IIIIP PPPHH | 0.588 | 0.722 | 0.518 | 0.567 | m6A site (Moderate confidence) |
|  |  | TGCCT GAACT TCCAC | HHHHH PPPPI IIIII |  |  |  |  |  |
|  |  | CACTG ATCAC TTGCC | IPPIP PPPPI IPPMM |  |  |  |  |  |
| 97 | 27469 | AGTTG GCGGG GCTCT | PPIIP PPMPP PPBPP | 0.595 | 0.617 | 0.676 | 0.628 | m6A site (High confidence) |
|  |  | CAGAT GAACT CCAAC | PPMMP PHHHH HPPMP |  |  |  |  |  |
|  |  | CCCTT GTTTC TCGCC | PPPII PPHHH HHPPI |  |  |  |  |  |
| 98 | 27523 | TGAGG CCCAC AAAAA | PPPPP PPPMM PPPPI | 0.753 | 0.66 | 0.547 | 0.666 | m6A site (High confidence) |
|  |  | TAGAG GGACT TGGCT | PPPII PPPPI PPPPI |  |  |  |  |  |
|  |  | CAAGA ACATG CAGTG | PPPPM MMMPP PPPPP |  |  |  |  |  |
| 99 | 27612 | GCAAG GGGAA AGGAA | PPIPP PPMMP PPPPP | 0.597 | 0.535 | 0.723 | 0.644 | m6A site (High confidence) |
|  |  | AAGGA AGACT GGGTC | PPPPM MPPPP PPPII |  |  |  |  |  |
|  |  | CTGGC CCCTG GCTTG | IIPPP PIIPP PBBPP |  |  |  |  |  |
| 100 | 28365 | AGCCC CCACA GCCTT | PPMPP PPPPP PIIPP | 0.657 | 0.59 | 0.626 | 0.641 | m6A site (High confidence) |
|  |  | GCAGA GGACA ACTAT | PPIPP PPPPP PBBPP |  |  |  |  |  |
|  |  | GGTAC ACAGG TCCAG | PIIPP PBPPP PPPPP |  |  |  |  |  |
| 101 | 28720 | TGCTA GTGGC AGAGC | MMPPP PIPPP HHHPP | 0.632 | 0.761 | 0.563 | 0.611 | m6A site (High confidence) |
|  |  | CTCTA GAACT GGGGC | PIPPP PMMPP PPPPP |  |  |  |  |  |
|  |  | CCTTC CCTGA AGCAG | PPPPP PPPPI PPPHH |  |  |  |  |  |
| 102 | 28847 | TTCCC CCAGG TGTTC | MMMMM PPPPP BPPPP | 0.734 | 0.74 | 0.652 | 0.701 | m6A site (Very high confidence) |
|  |  | GGCCT GGACT CCATC | PPPPP PPIPP PPBPP |  |  |  |  |  |
|  |  | ATGGG CAACA AGGAC | MMPPP PIPPP HHHHH |  |  |  |  |  |
| 103 | 28868 | GACTC CATCA TGGGC | PHHHP PPMPP IPPPP | 0.629 | 0.732 | 0.679 | 0.654 | m6A site (High confidence) |
|  |  | AACAA GGACC TGTGG | IPPPH HHHHH PPPIP |  |  |  |  |  |
|  |  | CCCCT GCTGC TGAGC | PPPIP PMMPP PPPPP |  |  |  |  |  |
| 104 | 29109 | AGTGC TAAAG AAGCT | PPPHH HHHHH HHPPP | 0.638 | 0.531 | 0.589 | 0.613 | m6A site (High confidence) |
|  |  | GCGCG GGACA GCTGA | PIPIP PPPPM PPIII |  |  |  |  |  |
|  |  | CGTGA CCCAT GACCT | PPPPH HHPPP PIIII |  |  |  |  |  |
| 105 | 30766 | TTCTG CATCC TGCCA | PPPBP PBPPP BPPPP | 0.702 | 0.714 | 0.665 | 0.688 | m6A site (Very high confidence) |
|  |  | CAGGC GGACT GTGCT | PPPPP PPBPP PPPPP |  |  |  |  |  |
|  |  | GCAGG CACAG ACAGG | PPPPP PMPPP PMMPP |  |  |  |  |  |
| 106 | 30784 | GCGGA CTGTG CTGCA | HHHHH PPPPP BBPPP | 0.482 | 0.543 | 0.651 | 0.553 | m6A site (Low confidence) |
|  |  | GGCAC AGACA GGGGA | PPIIP PPPMM MPPPP |  |  |  |  |  |
|  |  | CCATG GGGCC TCTTA | PPPPP PPPPP PIIII |  |  |  |  |  |
| 107 | 30791 | GTGCT GCAGG CACAG | PPPBB PPPPP IIPPP | 0.518 | 0.616 | 0.685 | 0.589 | m6A site (Moderate confidence) |
|  |  | ACAGG GGACC ATGGG | PMMMP PPPPP PPPPP |  |  |  |  |  |
|  |  | GCCTC TTAGC ACCGT | PPPPI IIIIP IIPPP |  |  |  |  |  |
| 108 | 30850 | CATTA CCACT CCCAG | MMMMM PPPPP PHHHH | 0.676 | 0.808 | 0.687 | 0.687 | m6A site (Very high confidence) |
|  |  | CGGGT GGACT CAACA | HPPPP PPMMM MMMMM |  |  |  |  |  |
|  |  | CTTGC AGCCA CCCTG | PPPPP PPHHH HHPPP |  |  |  |  |  |
| 109 | 30877 | ACACT TGCAG CCACC | MMMPP PPPPP HHHHH | 0.688 | 0.728 | 0.667 | 0.681 | m6A site (Very high confidence) |
|  |  | CTGCA GGACT TAAAT | PPPPP PPMMM MMMMM |  |  |  |  |  |
|  |  | CATTT TGTTC GGATG | MMMMM MMMMM PPPPP |  |  |  |  |  |
| 110 | 31904 | GGAGC ACAGG CAGCT | PPPPP PPPPM MPPPP | 0.852 | 0.672 | 0.852 | 0.843 | m6A site (Very high confidence) |
|  |  | GGATG AGACT TCCAA | PPIPP PPMMM PPPMM |  |  |  |  |  |
|  |  | ACCTG ACAGA TGTCA | MMPPP PPHHH HPPPP |  |  |  |  |  |
| 111 | 31988 | GATGT CCAGA AGAAT | PPPPI IIIPP PPPMM | 0.912 | 0.605 | 0.817 | 0.858 | m6A site (Very high confidence) |
|  |  | ATTCA GGACT TAACG | MMMMM PPPPP PPBBP |  |  |  |  |  |
|  |  | GCTCC AGGAT TTTAA | PPPII IIPPI IIPPP |  |  |  |  |  |
| 112 | 32021 | CCAGG ATTTT AACAA | IIIIP PIIIP PPPPP | 0.853 | 0.478 | 0.784 | 0.806 | m6A site (Very high confidence) |
|  |  | AAGCA AGACT GTTGC | PPIPP MMMPP PPPPP |  |  |  |  |  |
|  |  | TCAAA TCTAT TCAGA | PIIII PPPHH HHPPP |  |  |  |  |  |
| 113 | 32161 | GGTTC CATGC CTGAG | PPPII IIIPP PPPMP | 0.828 | 0.466 | 0.714 | 0.764 | m6A site (Very high confidence) |
|  |  | GGTGG AGACT AAGCC | PPPPP PPPII IIPPI |  |  |  |  |  |
|  |  | CTGTC GAGAC ACTTG | IPPPH HHHPP PIIIP |  |  |  |  |  |
| 114 | 32230 | GGGCT GGACC TATGT | HPPPP PPMMM MMMPP | 0.595 | 0.619 | 0.586 | 0.593 | m6A site (Moderate confidence) |
|  |  | CCTAA GGACA CACTA | PPPPP PPMMM MMMMM |  |  |  |  |  |
|  |  | ATCGA ACTAT GAACT | MMMPP PPPIP PHHHH |  |  |  |  |  |
| 115 | 32243 | GTCCT AAGGA CACAC | PPPPP PPPPM MMMMM | 0.882 | 0.718 | 0.86 | 0.865 | m6A site (Very high confidence) |
|  |  | TAATC GAACT ATGAA | MMMMM PPPPP IPPHH |  |  |  |  |  |
|  |  | CTACA AAGCT TCTAT | HHHPP IPPBP PPMMM |  |  |  |  |  |
| 116 | 32250 | GGACA CACTA ATCGA | PPMMM MMMMM MMMPP | 0.852 | 0.5 | 0.875 | 0.843 | m6A site (Very high confidence) |
|  |  | ACTAT GAACT ACAAA | PPPIP PHHHH HPPIP |  |  |  |  |  |
|  |  | GCTTC TATCC CAGGA | PBPPP MMMMM PPPPP |  |  |  |  |  |
| 117 | 32397 | AATTA ATCTT TCTTT | IIIPP PHHHH HHHPP | 0.771 | 0.291 | 0.826 | 0.769 | m6A site (Very high confidence) |
|  |  | ACCTG AGACC AGTTG | PIIPP PPIII IPPPP |  |  |  |  |  |
|  |  | GGAGC ACTGG AGTGC | PPMPP PPHHH HPPPP |  |  |  |  |  |
