# Supplementary figures and images for: Methylation Drives SLC2A1 Transcription and Ferroptosis Process Decreasing Autophagy Pressure in Colon Cancer
Source: J Oncol. 2022 Aug 27;2022:9077424. doi: 10.1155/2022/9077424 (PMC9440784; doi:10.1155/2022/9077424)

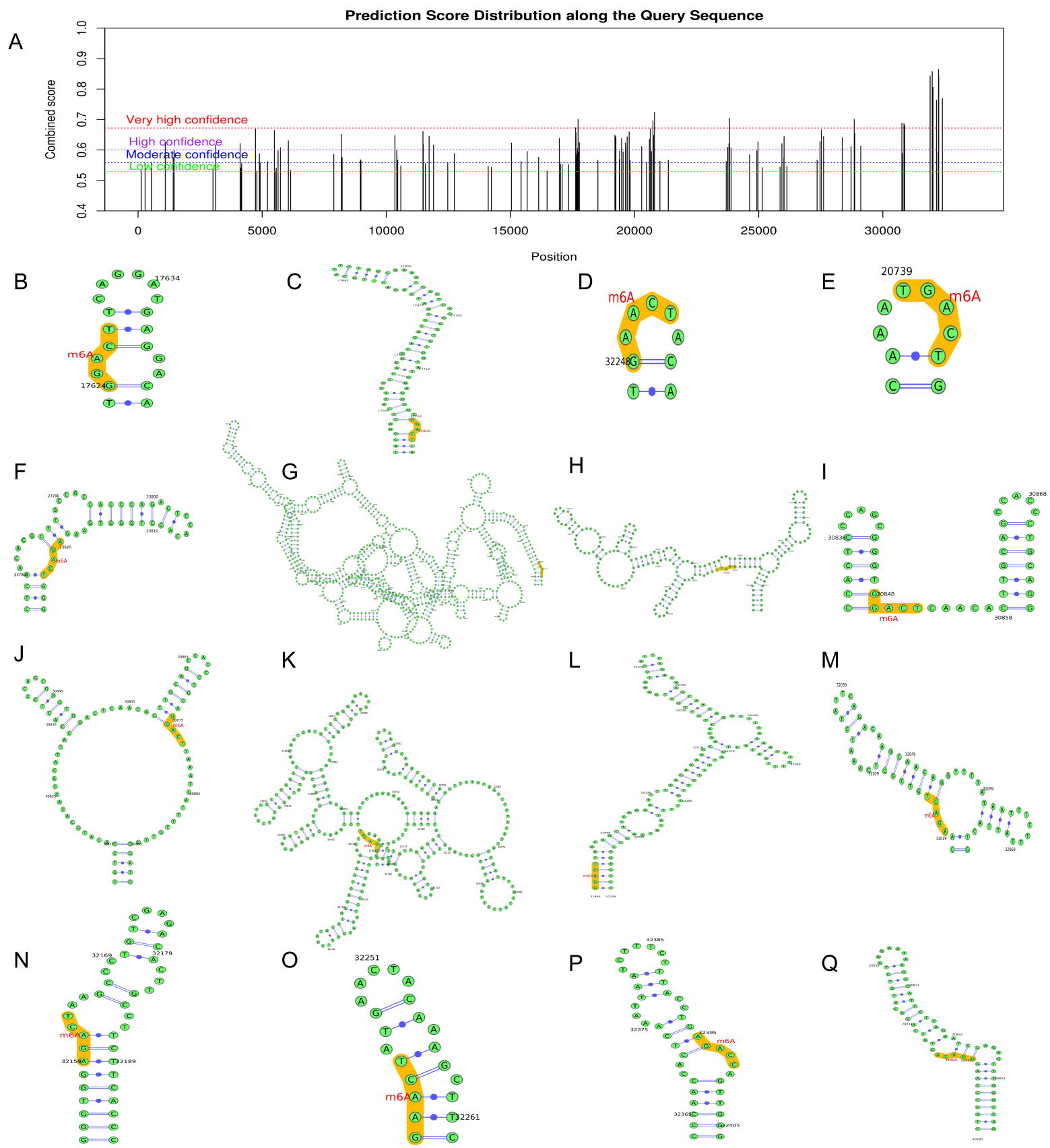

Supplement: Supplementary Materials — Supplementary Figure 1. Intersection of differential genes and autophagy genes. A: there were 113 intersecting genes of common downregulating genes and autophagy genes. B: there were 73 intersecting genes of coupregulated genes and autophagy genes. C: forest map is shown. Supplementary Figure 2. The m6a site on the SLC2A1 sequence and its secondary RNA structure are predicted. A: distribution map of m6a prediction sites. B–Q: secondary RNA structure with very high-confidence methylation sites. Supplementary Figure 3. Comprehensive exploration of the methylation probes of SLC2A1. A: heat map of SLC2A1 in MEXPRESS is illustrated. B: heat map of SLC2A1 in MethSurv is shown. C–F: methylation probes of clinical significance in SLC2A1 are depicted. Supplementary Figure 4. Analysis of GEO data using EcoTyper. A: the row of the heat map corresponds to cell state, the column corresponds to sample, and the colour corresponds to cell state abundance. The colour bar on the left represents the state of each cell, the cancer ecotype it forms, its cell type, and its ID. The top colour bar indicates the cancer ecotype with the highest abundance in each sample. B–M: a heat map is shown on the left, which depicts the cell type-specific expression of cell state characteristics of genes in TCGA cancer samples for reference. The right side shows the heat map of gene expression of cell state characteristics in GEO data. N: classification diagram of cell interaction state is shown. Supplementary Figure 5. Stacked histogram of immune infiltration scores. A-B: distribution of immune scores of GSE23878 and GSE113513 in CIBERSORTx are illustrated. C–E: immune infiltration scores of GSE 10972, GSE23878, and GSE113513 in xCell are shown. Supplementary Figure 6. Biological functions affected by SLC2A1 and correlation with specific proteins. A: Effects of SLC2A1 can be seen in GeneMANIA. B: correlation between SLC2A1 expression levels and methylase, autophagy and ferroptosis proteins in COAD is s [file 9077424.f1.zip › sup-figures and sup-tables/suppl-fig2.pdf]

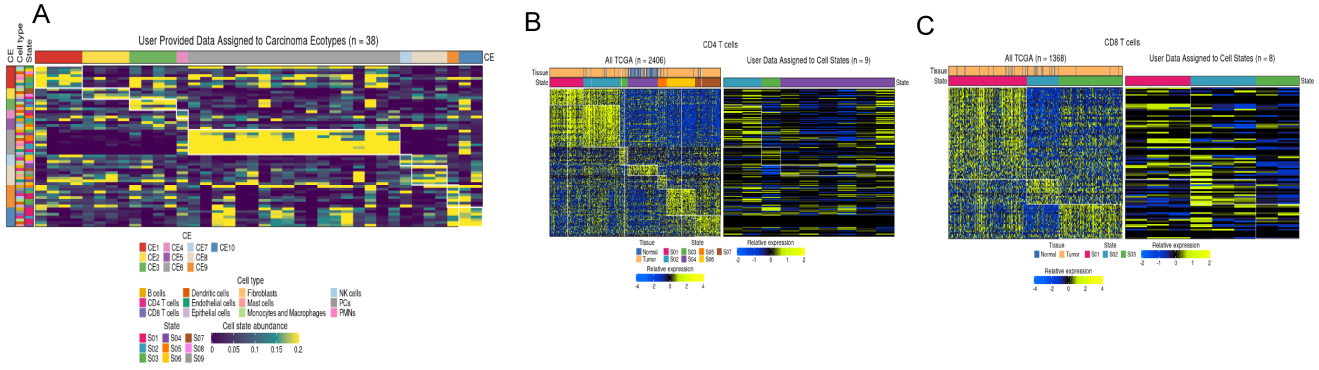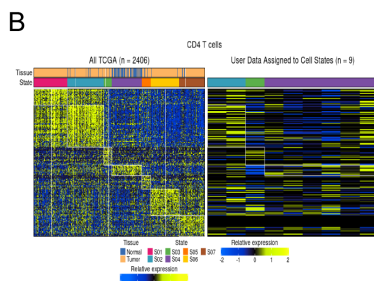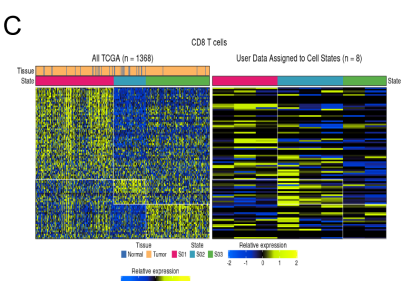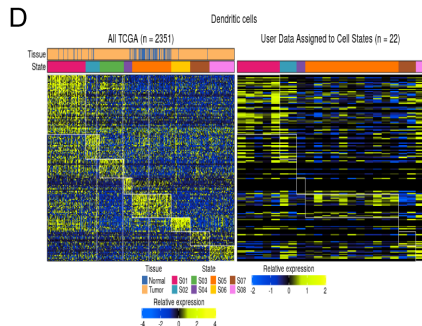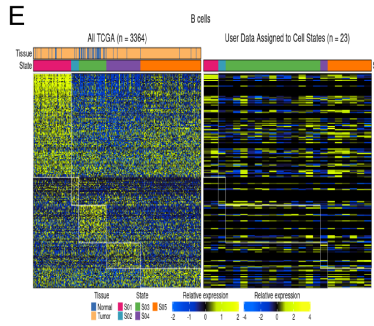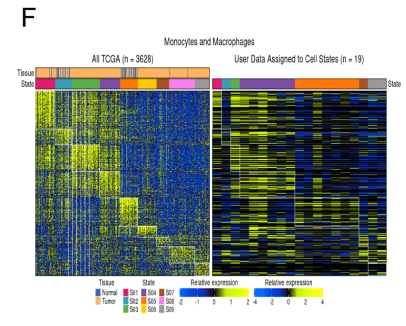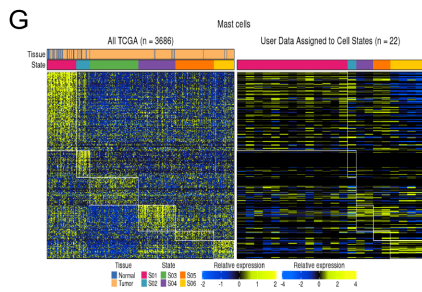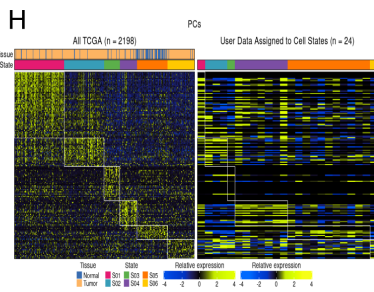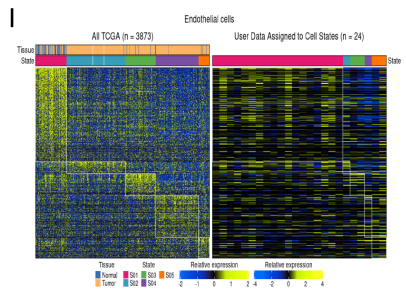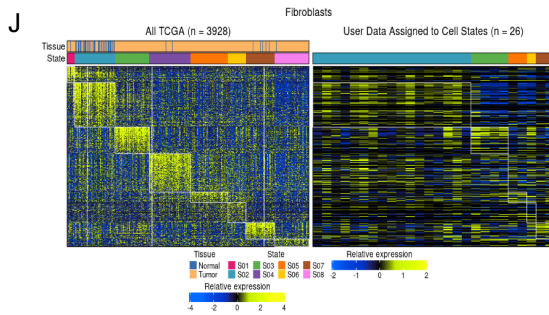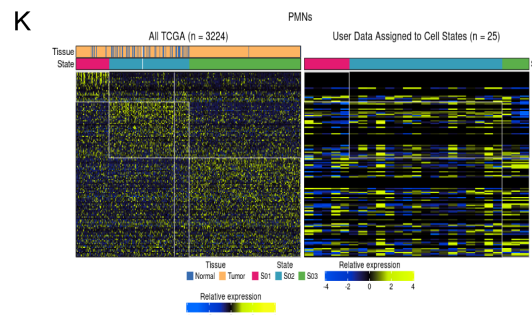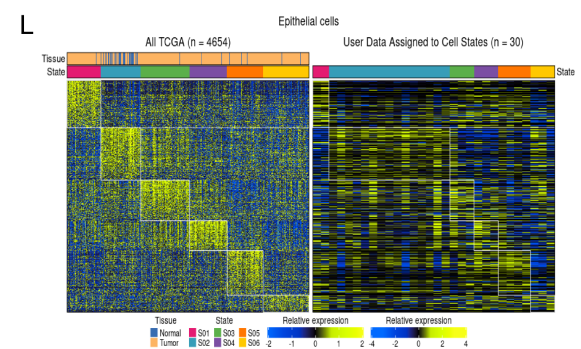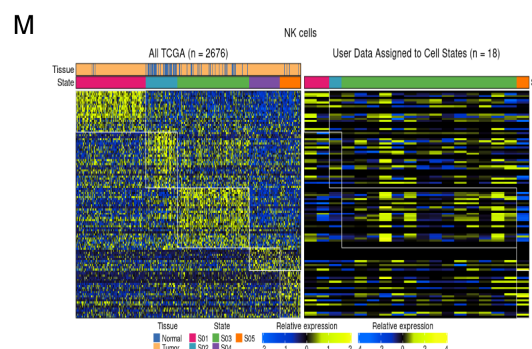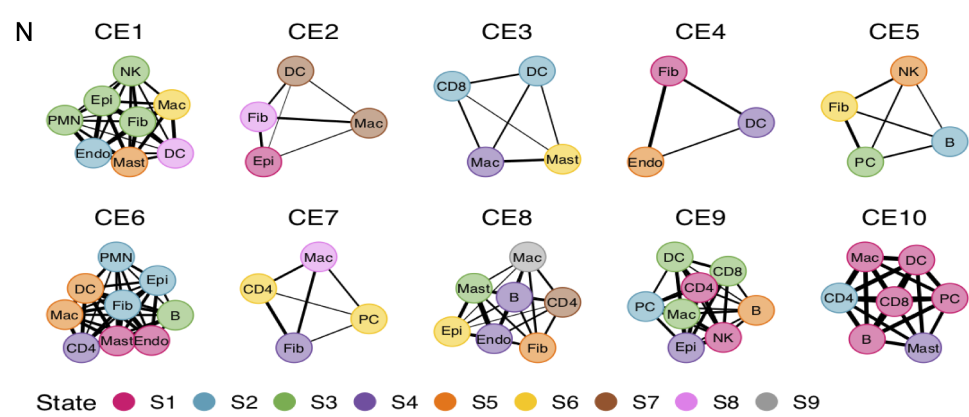

Supplement: Supplementary Materials — Supplementary Figure 1. Intersection of differential genes and autophagy genes. A: there were 113 intersecting genes of common downregulating genes and autophagy genes. B: there were 73 intersecting genes of coupregulated genes and autophagy genes. C: forest map is shown. Supplementary Figure 2. The m6a site on the SLC2A1 sequence and its secondary RNA structure are predicted. A: distribution map of m6a prediction sites. B–Q: secondary RNA structure with very high-confidence methylation sites. Supplementary Figure 3. Comprehensive exploration of the methylation probes of SLC2A1. A: heat map of SLC2A1 in MEXPRESS is illustrated. B: heat map of SLC2A1 in MethSurv is shown. C–F: methylation probes of clinical significance in SLC2A1 are depicted. Supplementary Figure 4. Analysis of GEO data using EcoTyper. A: the row of the heat map corresponds to cell state, the column corresponds to sample, and the colour corresponds to cell state abundance. The colour bar on the left represents the state of each cell, the cancer ecotype it forms, its cell type, and its ID. The top colour bar indicates the cancer ecotype with the highest abundance in each sample. B–M: a heat map is shown on the left, which depicts the cell type-specific expression of cell state characteristics of genes in TCGA cancer samples for reference. The right side shows the heat map of gene expression of cell state characteristics in GEO data. N: classification diagram of cell interaction state is shown. Supplementary Figure 5. Stacked histogram of immune infiltration scores. A-B: distribution of immune scores of GSE23878 and GSE113513 in CIBERSORTx are illustrated. C–E: immune infiltration scores of GSE 10972, GSE23878, and GSE113513 in xCell are shown. Supplementary Figure 6. Biological functions affected by SLC2A1 and correlation with specific proteins. A: Effects of SLC2A1 can be seen in GeneMANIA. B: correlation between SLC2A1 expression levels and methylase, autophagy and ferroptosis proteins in COAD is s [file 9077424.f1.zip › sup-figures and sup-tables/suppl-fig4.pdf]

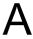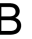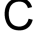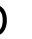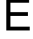

Supplement: Supplementary Materials — Supplementary Figure 1. Intersection of differential genes and autophagy genes. A: there were 113 intersecting genes of common downregulating genes and autophagy genes. B: there were 73 intersecting genes of coupregulated genes and autophagy genes. C: forest map is shown. Supplementary Figure 2. The m6a site on the SLC2A1 sequence and its secondary RNA structure are predicted. A: distribution map of m6a prediction sites. B–Q: secondary RNA structure with very high-confidence methylation sites. Supplementary Figure 3. Comprehensive exploration of the methylation probes of SLC2A1. A: heat map of SLC2A1 in MEXPRESS is illustrated. B: heat map of SLC2A1 in MethSurv is shown. C–F: methylation probes of clinical significance in SLC2A1 are depicted. Supplementary Figure 4. Analysis of GEO data using EcoTyper. A: the row of the heat map corresponds to cell state, the column corresponds to sample, and the colour corresponds to cell state abundance. The colour bar on the left represents the state of each cell, the cancer ecotype it forms, its cell type, and its ID. The top colour bar indicates the cancer ecotype with the highest abundance in each sample. B–M: a heat map is shown on the left, which depicts the cell type-specific expression of cell state characteristics of genes in TCGA cancer samples for reference. The right side shows the heat map of gene expression of cell state characteristics in GEO data. N: classification diagram of cell interaction state is shown. Supplementary Figure 5. Stacked histogram of immune infiltration scores. A-B: distribution of immune scores of GSE23878 and GSE113513 in CIBERSORTx are illustrated. C–E: immune infiltration scores of GSE 10972, GSE23878, and GSE113513 in xCell are shown. Supplementary Figure 6. Biological functions affected by SLC2A1 and correlation with specific proteins. A: Effects of SLC2A1 can be seen in GeneMANIA. B: correlation between SLC2A1 expression levels and methylase, autophagy and ferroptosis proteins in COAD is s [file 9077424.f1.zip › sup-figures and sup-tables/suppl-fig5.pdf]
